# Nicotinamide Metabolism Constrains Memory CD8^+^ T Cell Formation Through a Putative HS1BP3‐SIRT1‐FOXO3‐BCL6 Axis

**DOI:** 10.1002/advs.77284

**Published:** 2026-08-21

**Authors:** Siyang Wang, Xiaoqian Fan, Yifan Huang, Chang Yao, Jixing Fan, Yu Ping, Xuan Zhao, Congcong Li, Chunyi Shen, Jiqi Shan, Jinyan Liu, Shengdian Wang, Zhen Zhang, Yi Zhang

**Affiliations:** ^1^ Biotherapy Center and Cancer Center The First Affiliated Hospital of Zhengzhou University Zhengzhou Henan China; ^2^ Zhongyuan Cell Therapy and Immunotherapy Laboratory Henan Academy of Innovations in Medical Science Zhengzhou China; ^3^ National Laboratory of Biomacromolecules Institute of Biophysics Chinese Academy of Sciences Beijing China; ^4^ School of Life Sciences Zhengzhou University Zhengzhou Henan China; ^5^ School of Public Health Zhengzhou University Zhengzhou Henan China; ^6^ State Key Laboratory of Metabolic Dysregulation & Prevention and Treatment of Esophageal Cancer Tianjian Laboratory of Advanced Biomedical Sciences Academy of Medical Sciences Zhengzhou University Zhengzhou China

**Keywords:** antitumor response, CD8^+^ T cell, memory, nicotinamide metabolism

## Abstract

Memory T cells exhibit long‐term persistence, a defining feature that underpins durable clinical responses to adoptive immunotherapies. The mechanisms that integrate metabolic cues with transcriptional control of memory fate remain undetermined. Here, we identify HS1‐binding protein 3 (HS1BP3) is preferentially expressed in memory CD8^+^ T cells. HS1BP3 deficiency reduced memory‐associated gene expression in CD8^+^ OT‐1 T cells following *Listeria monocytogenes–*ovalbumin infection and impaired antitumor responses. Loss of HS1BP3 induces metabolic reprogramming characterized by reduced oxidative phosphorylation (OXPHOS) and altered nicotinamide metabolism, accompanied by increased NAD^+^ and nicotinamide metabolite 1‐methylnicotinamide (MNAM) abundance. HS1BP3 interacted with Sirtuin 1 (SIRT1), and its deficiency is associated with increased SIRT1 activity, enhanced Forkhead box O3 (FOXO3) signaling, and reduced expression of memory‐associated transcription factor B cell lymphoma 6 (BCL6). Moreover, accumulation of MNAM impairs the antitumor activity of CD8^+^ T cells. Importantly, elevated levels of HS1BP3 drive chimeric antigen receptor (CAR) ‐T cells towards a memory phenotype and improve tumor control. Collectively, our findings identify HS1BP3 as a regulator of CD8^+^ T cell memory and indicate that its effects are associated with alterations in nicotinamide metabolism and the SIRT1–FOXO3–BCL6 signaling axis. These observations support the therapeutic potential of HS1BP3‐engineered CAR‐T cells across solid tumors.

## Graphical abstract

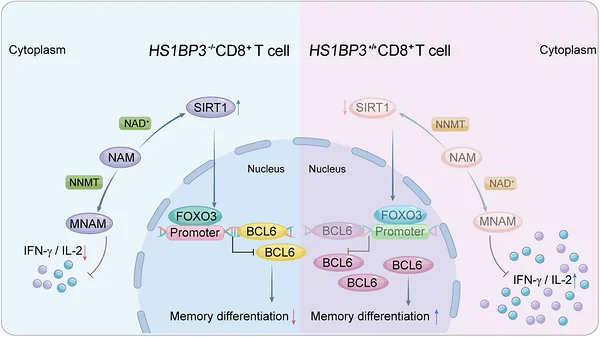

**Abbreviations**
BCL6: B‐cell lymphoma 6
CAR: chimeric antigen receptor
DEGs: differentially expressed genes
DEPs: differentially expressed proteins
DN: double negative
DP: double positive
FOXO: forkhead box O
HS1BP3: HS1 binding protein 3
KO: knockout
LCMV: lymphocytic choriomeningitis virus
LLC‐OVA: Lewis lung carcinoma‐ovalbumin
Lm‐OVA: Listeria monocytogenes expressing ovalbumin
MNAM: 1‐methylnicotinamide
NAD^+^
: nicotinamide adenine dinucleotide
NAMPT: nicotinamide phosphoribosyltransferase
OCR: oxygen consumption rate
OXPHOS: oxidative phosphorylation
Tcf‐1: T‐cell factor 1
T_cm_
: central memory T cells
T_eff_
: effector T cells
T_em_
: effector memory T cells
T_m_
: memory T cells
SP: single positive
WT: wild type

## 1 Introduction

CD8^+^ memory T (T_m_) cells represent vital effectors in immune responses against tumor and viral infections, and are characterized by their persistence, heightened cytolytic activity, and self‐renewal capacity [1, 2, 3]. CD8^+^ T_m_ cells are generally subdivided into central memory cells (T_cm_) and effector memory cells (T_em_), distinguished by phenotypic signatures and functional attributes. Compared to effector T cells (T_eff_) and T_em_, T_cm_ cells exhibit a superior capacity during secondary challenge [4]. Accumulating clinical evidence indicates that higher frequencies of T_m_ cells are correlated with improved outcomes among patients undergoing immunotherapy [5, 6], and the adoptive transfer of memory‐enriched chimeric antigen receptor (CAR)‐T cell products facilitates prolonged in vivo longevity and durable tumor suppression [7, 8]. Importantly, identifying the key regulators that govern T_m_ cell differentiation has emerged as a promising strategy to enhance the clinical efficacy of solid tumor immunotherapy.

Transcription factors, including *TCF‐1* [9, 10], *BCL‐6* [11], and *FOXO1* [12], promote gene expression programs that are characteristic of long‐lived memory cells. In addition, metabolic interventions targeting metabolites or key enzymes modulate memory fitness and homeostatic persistence of T cells in the tumor microenvironment [13, 14, 15]. However, the mechanisms underlying the interplay between metabolic molecules and transcription factors during T_m_ cell differentiation remain unclear. Recently, nicotinamide adenine dinucleotide (NAD^+^) and its metabolites have emerged as pivotal determinants in T_m_ cell differentiation and function [16, 17]. Furthermore, NAD^+^ functions as a redox coenzyme and serves as a critical substrate for sirtuins, including SIRT1, which regulates transcription factors and chromatin remodeling [18]. However, activation of the NAD^+^‐ SIRT1‐ CD38 axis has been implicated as a major driver of CD8^+^ T cell dysfunction in the tumor microenvironment [19]. Correspondingly, the NAD^+^ metabolism product 1‐methylnicotinamide (MNAM) restrains T cell‐mediated antitumor responses [20]. Hence, the mechanisms by which NAD^+^ metabolism specifically engages its signaling cascade components require in‐depth investigation during T_m_ cell lineage commitment.

HS1 binding protein 3 (HS1BP3) is an important modulator of immunological homeostasis and a potential inhibitor of autophagy [21]. Notably, SIRT1 is an NAD^+^‐dependent deacetylase, that regulates autophagy through multiple pathways, including the deacetylation of transcription factors containing forkhead box Os (FOXOs) and autophagy‐related genes [22]. Although HS1BP3 is associated with prognosis in hepatocellular carcinoma [23], the mechanism by which HS1BP3 regulates into NAD^+^‐ SIRT1 pathways to orchestrate CD8^+^ T cell memory differentiation is unclear.

Herein, HS1BP3 was first characterized as a differentially expressed molecule between CD8^+^ T_eff_ and T_m_ cells using proteomic analysis and transcriptome data, which were verified using GEO dataset. This study further demonstrated that loss of HS1BP3 impairs the development of CD8^+^ OT‐1 T_cm_ cells (CD44^+^CD62L^+^) and diminishes the recall response of antigen‐specific CD8^+^ T cells. Mechanistically, HS1BP3‐deficient CD8^+^ T cells exhibit enhanced nicotinamide metabolism and accumulation of its metabolite MNAM. Increased nicotinamide metabolism elevates NAD^+^ levels, which promotes SIRT1 activity and subsequently activates the FOXO3 signaling pathway. In parallel, MNAM accumulation impairs the antitumor activity of CD8^+^ OT‐1 T cells. Conversely, increased HS1BP3 expression reduced SIRT1 and FOXO3 expression and decreased MNAM levels in CAR‐T cells. These findings identify HS1BP3 as a key regulator of memory programming in CD8^+^ T cells and indicate that the modulation of HS1BP3 may bolster the clinical performance of CAR‐T cell therapy in solid tumors.

## 2 Results

### 2.1 HS1BP3 Expression Is Enriched in Memory CD8^+^ T Cells

To determine the key molecules involved in regulating T_m_ cell differentiation, CD45.1^+^ CD8^+^ OT‐1 T cells were engrafted into CD45.2^+^ C57BL/6 mice, following infection with *Listeria monocytogenes* expressing ovalbumin (*Lm*‐OVA), as previously described [24]. Effector (day 7) and memory (day 30) CD8^+^ T cells were successfully separated after the initial infection (Figure 1A). Proteomics analysis comparing T_eff_ and T_m_ cells identified 759 differentially expressed proteins (DEPs), as shown in the volcano plot (Figure 1A). Hs1bp3 showed the greatest differential expression and mitochondrial enrichment in T_m_ cells (Figure 1A,B). In parallel, transcriptomic analysis of differentially expressed genes (DEGs) revealed enrichment of oxidative phosphorylation (OXPHOS) in T_m_ cells (Figure 1B). Integrated transcriptomic and proteomic analyses using Venn diagrams and heatmaps further identified Hs1bp3 as a significantly upregulated gene and protein in T_m_ cells (Figure 1C,D). In the *lymphocytic choriomeningitis virus* (LCMV) infection model, this finding was further verified using the RNA‐sequencing (RNA‐seq) data from CD8^+^ T cell subsets (Figure 1E). The expression level of HS1BP3 was also detected in T_eff_ and T_m_ cells from mouse and human samples. These results demonstrated that HS1BP3 was more highly expressed in T_m_ cells than in T_eff_ cells (Figure 1F,G). Thus, Hs1bp3 is enriched in memory CD8^+^ T cells.

**FIGURE 1:**
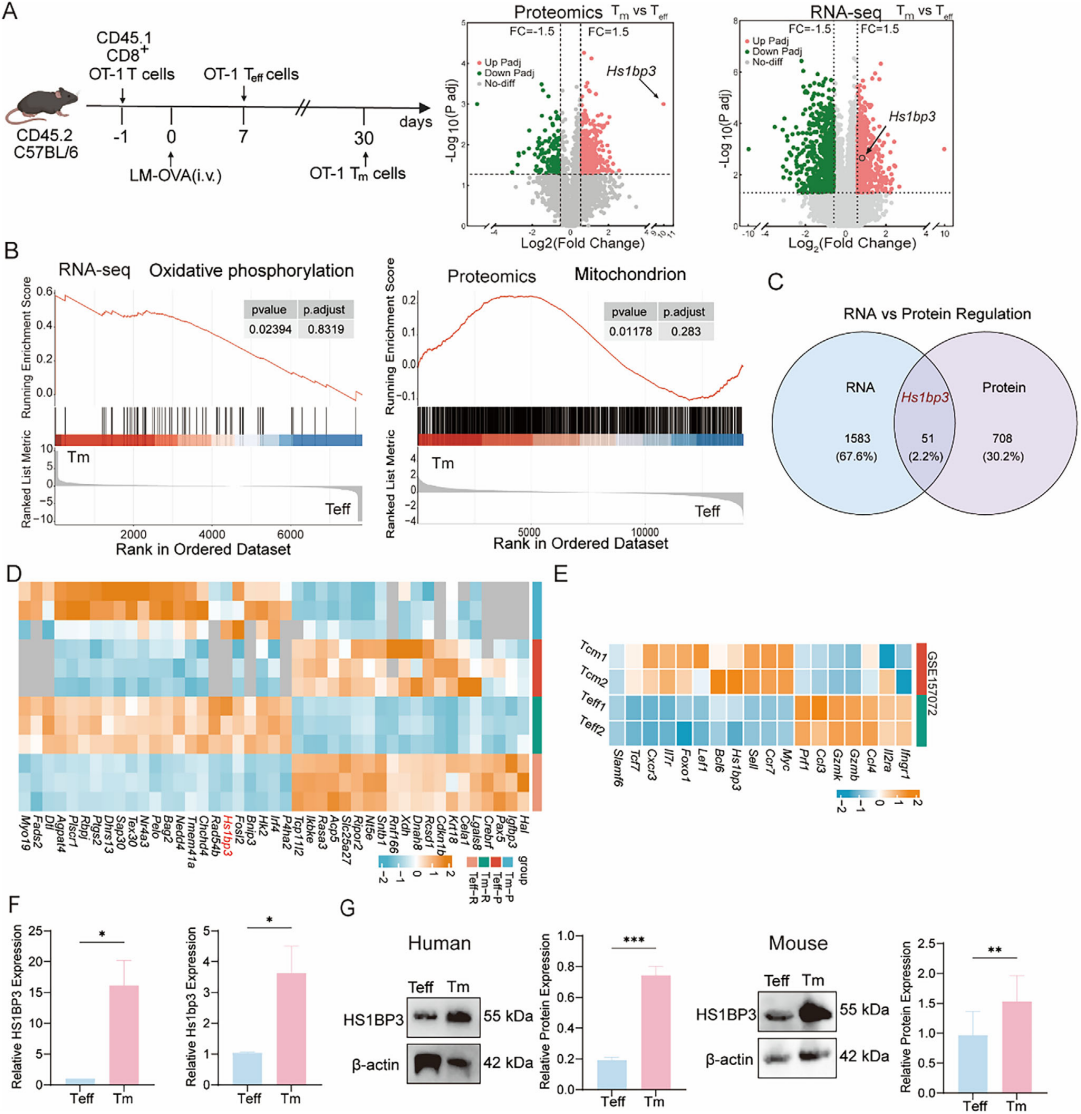
HS1BP3 is upregulated in memory CD8^+^ T cells. (A) Proteomics and RNA‐seq analyses of CD8^+^ OT‐1 T_eff_ and T_m_ cells following *Lm*‐OVA infection were performed to identify differentially expressed proteins and genes (*n* = 5 biological replicates). (B) GSEA highlighting pathways related to oxidative phosphorylation and mitochondrion in T_m_ cells. (C) Venn analysis showed the differentially expressed genes and proteins. (D) Heatmap showing the differentially expressed genes and proteins. (E) Heatmap showing Hs1bp3 expression in T_cm_ and T_eff_ cells from GSE157072 data. (F‐G) Human and mouse CD8^+^ T cells were induced T_eff_ or T_m_ cells. HS1BP3 expression was measured using qRT‐PCR (F) and western blot (G) (*n* = 3 biological replicates). This experiment was independently performed three times. Data are expressed as mean ± SEM. Statistical significance was evaluated by unpaired two‐tailed Student's t‐test(F‐G). *, *P* < 0.05; **, *P* < 0.01; ***, *P* < 0.001.

### 2.2 HS1BP3 Deletion Impairs the Memory Formation of CD8^+^ T Cells

To further investigate how HS1BP3 influences CD8^+^ T cell differentiation, we generated CD45.1^+^ *Hs1bp3*‐knockout OT‐1 mice. Western blot analysis indicated the absence of HS1BP3 in CD8^+^ T cells compared to controls (Figure S1A). The study showed that the proportions of CD4^−^CD8^−^ double‐negative (DN) cells, CD4^+^CD8^+^ double‐positive (DP) cells, CD4^+^CD8^−^ and CD4^−^CD8^+^ single‐positive (SP) cells, within the total thymocyte pools and percentage of different stages of DN (DN1‐4) cells showed no notable difference between *Hs1bp3* ^+/+^ and *Hs1bp3* ^−/−^ OT‐1 mice (Figure S1B). CD4^+^ and CD8^+^ T cell ratios were also detected in the lymph nodes; however, this did not change in *Hs1bp3* ^+/+^or *Hs1bp3* ^−/−^ OT‐1 mice (Figure S1B). Consistent results were verified in global Hs1bp3 knockout (KO) and wild‐type (WT) mice (Figure S1C), indicating that the ablation of Hs1bp3 does not affect thymus T cell development. The frequency of CFSE^low^CD8^+^ T cells was not substantially different between the two groups (Figure S1D). Interestingly, the frequencies of CD44^+^CD62L^±^ cells were markedly downregulated in CD8^+^ T cells from *Hs1bp3* ^−/−^ OT‐1 mice (Figure S1E). To characterize the effect of Hs1bp3 on T_m_ cell differentiation, *Hs1bp3* ^−/−^ or *Hs1bp3* ^+/+^ CD8^+^ OT‐1 T cells were adoptively infused into CD45.2^+^ recipients, followed by *Lm*‐OVA. On day 30 post‐challenge, *Hs1bp3* ^−/−^ CD8^+^ OT‐1 T cells showed a substantial decline in both the proportion and absolute count of CD44^+^CD62L^+/−^ cells (Figure 2A,B and Figure S1F). Accordingly, the splenic proportion of memory‐precursor T_eff_ cells (MPEC, CD127^+^KLRG1^−^) was reduced in *Hs1bp3^−/^* ^−^ CD8^+^ OT‐1 T cells relative to that in the control group, which preferentially differentiated into long‐lived T_cm_ cells [25]. Furthermore, the frequency of short‐lived T_eff_ cells (SLEC, CD127^−^KLRG1^+^) increased in the *Hs1bp3* ^−/−^ group (Figure S2A). These cells also showed decreased expression of the memory‐related marker T‐cell factor 1 (Tcf‐1; Figure 2C and Figure S1F), Eomes and Bcl6 (Figure S2B), and elevated Blimp‐1 and T‐bet levels (Figure S2C). These results revealed that Hs1bp3 orchestrates the development of CD8^+^ memory cell subsets. T_m_ cells display a metabolic profile characterized by OXPHOS [26]. In this study, we observed that the level of mitochondrial OXPHOS proteins (I‐NDUFB8, II‐SDHB, III‐UQCRC2, and V‐ATP5A1) was substantially decreased in *Hs1bp3* ^−/−^ CD8^+^ OT‐1 T cells (Figure 2D), as were the oxygen consumption rate (OCR) and spare respiratory capacity (Figure 2E). In addition, the expression of memory‐related genes (*IL7r*, *Sell, Tcf‐7*, *Lef1* and *Bcl6*) decreased in Hs1bp3‐deficient CD8^+^ T cells (Figure 2F). However, how HS1BP3 deficiency affects the recall response of memory CD8^+^ T cells remains unclear. Next, CD44^+^CD62L^+^ OT‐1 T cells were sorted from primary hosts infected with *Lm*‐OVA, and subsequently engrafted into CD45.2^+^ recipients, and challenged with *Lm*‐OVA (Figure 2G). The recall potential of Hs1bp3‐deficient T_m_ cells was markedly hampered (Figure 2H–J). These data indicated that Hs1bp3 ablation limited both memory cell formation and secondary expansion potential.

**FIGURE 2:**
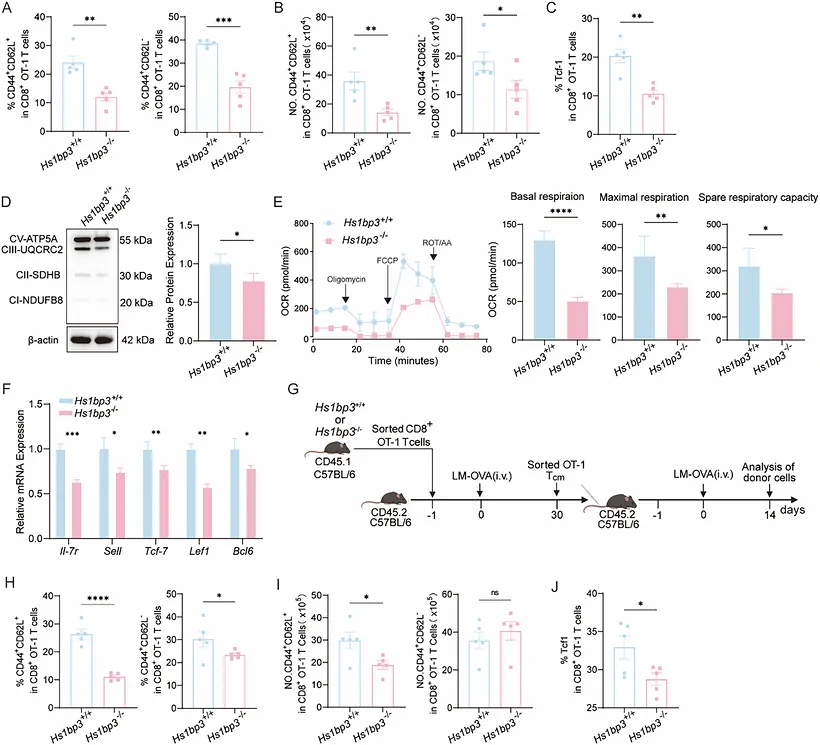
HS1BP3 deficiency disrupts memory CD8^+^ T cell differentiation in the *Lm*‐OVA challenge model. CD45.1^+^ *Hs1bp3* ^+/+^ or *Hs1bp3* ^−/−^ CD8^+^ OT‐1 T cells were infused into CD45.2^+^ recipients following *Lm‐*OVA infection (*n* = 5 biological replicates). (A, B) Proportions and numbers of CD44^+^CD62L^+^ and CD44^+^CD62L^−^ subsets on day 30 via flow cytometry. (C) The frequency of Tcf‐1^+^ T cells as evaluated through FACS. (D) Immunoblot (left) and quantification of mitochondrion OXPHOS proteins (I‐NDUFB8, II‐SDHB, III‐UQCRC2, and V‐ATP5A1) in CD8^+^ T cells from *Hs1bp3* ^+/+^ or *Hs1bp3* ^−/−^. (E) OCR of *Hs1bp3* ^+/+^ or *Hs1bp3* ^−/−^ CD8^+^ T cells (left). Quantification of basal respiration, maximal respiration, and spare respiration capacity of the cells (right). FCCP, Carbonyl cyanide 4‐(trifluoromethoxy) phenylhydrazone; Rot/AA, rotenone and antimycin A (*n* = 5 biological replicates). (F) The transcriptional abundance of essential memory‐associated genes in CD8^+^ T cells was quantified via qRT‐PCR (*n* = 3 biological replicates). This experiment was independently performed three times. (G) Experimental design of the recall response model. The CD45.2^+^ mice receiving CD45.1^+^ *Hs1bp3* ^+/+^ or *Hs1bp3* ^−/−^ CD8^+^ OT‐1 T cells were infected with LM‐OVA to generate primary memory populations. On day 30, the donor cells were sorted and transferred into CD45.2^+^ hosts within secondary *Lm*‐OVA challenge (*n* = 5 biological replicates). (H, I) Frequencies and numbers of CD44^+^CD62L^+^ and CD44^+^CD62L^−^ subsets on day 44 post‐secondary *Lm*‐OVA challenge. (J) Tcf‐1 levels in CD8^+^ T cells. Values are expressed as mean ± SEM. Statistical significance by unpaired two‐tailed Student's t‐test (A–D, F, and H‐J). *, *P* < 0.05; **, *P* < 0.01; ***, *P* < 0.001; ****, *P* < 0.0001.

### 2.3 Loss of HS1BP3 Impairs the Antitumor Function of CD8^+^ T Cells

The regulatory involvement of HS1BP3 in orchestrating the antitumor functions of CD8^+^ T cells was further elucidated. In a subcutaneous Lewis lung carcinoma‐ovalbumin (LLC‐OVA) cell implantation model, *Hs1bp3* ^−/−^ OT‐1 mice exhibited accelerated tumor growth (Figure S3A,B) and shorter survival (Figure S3C). On day 14, tumor‐infiltrating *Hs1bp3* ^−/−^ CD8^+^ T cells contained a lower frequency of CD44^+^CD62L^+^ cells than those in *Hs1bp3* ^+/+^ OT‐1 mice, as well as a lower number per milligram of tumor (Figure S3D). Moreover, the reduced IFN‐γ protein levels demonstrated that Hs1bp3‐deficiency also attenuated the effector competence of CD8^+^ T cells (Figure S3D). The sustained immunosurveillance capacity afforded by HS1BP3 was further substantiated in a resection‐rechallenge model, which demonstrated that the memory‐centric T cell reservoir was sufficient to thwart the establishment of secondary tumors independent of the primary lesion [27, 28]. Similar results were observed in the *Hs1bp3* ^−/−^ tumor rechallenge model (Figure S3E–H). To ascertain the contribution of CD8^+^ T cells to Hs1bp3‐mediated immunosurveillance. CD8^+^ T cells were depleted in vivo and challenged with LLC‐OVA lung cancer (Figure S4A,B), and the results exhibited that tumor volume and survival time did not differ between *Hs1bp3* ^−/−^ and *Hs1bp3* ^+/+^ OT‐1 mice (Figure S4C,D).

To validate this hypothesis, we adoptively infused *Hs1bp3* ^−/−^ or *Hs1bp3* ^+/+^ CD8^+^ OT‐1 T cells into LLC‐OVA tumor‐bearing hosts (Figure 3A). Recipients of *Hs1bp3* ^−/−^ CD8^+^ T cells developed larger tumors and had shorter survival times than those in the control group (Figure 3B–D). Moreover, Hs1bp3 deletion reduced the proportion and absolute count of memory CD8^+^ OT‐1 T cells (CD44^+^CD62L^+^ and CD44^+^CD62L^−^ cells) in the tumors (Figure 3E,F). Intratumoral *Hs1bp3* ^−/−^ CD8^+^ OT‐1 T cells also exhibited low levels of Tcf‐1 (Figure 3G), Bcl6, and Ly108, whereas the PD‐1^+^ Tim3^+^ and TOX^+^ T cells were increased (Figure S5A,B). Concurrently, pro‐inflammatory cytokines including IFN‐γ, Granzyme B, and IL‐2 were decreased in *Hs1bp3* ^−/−^ CD8^+^ OT‐1 T cells (Figure 3H). Further validation of the Hs1bp3‐mediated antitumor response was conducted in the tumor cell rechallenge model (Figure 3I). Deficiency of Hs1bp3 during T_m_ cell formation accelerated tumor growth in the LLC‐OVA tumor model (Figure 3J,K), which also led to a reduction in T_m_ cells (Figure 3L–N) and cytokine production (Figure 3O). A representative gating strategy is shown in Figure S6. Altogether, these findings establish that Hs1bp3 is vital for the optimal induction of CD8^+^ T cell memory, and its deficiency resulting in impaired immune surveillance and blunted recall potential.

**FIGURE 3:**
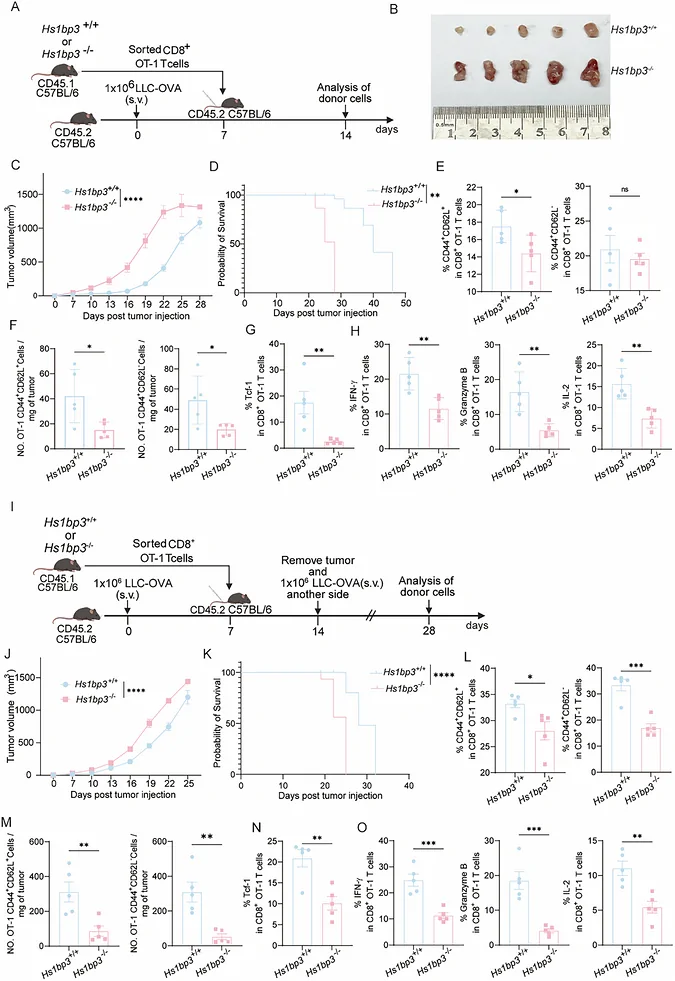
HS1BP3 deficiency inhibits anti‐tumor response of CD8^+^ T cells. (A) Diagram of cellular infusion of *Hs1bp3* ^+/+^ or *Hs1bp3* ^−/−^ CD8^+^ OT‐1 T cells into LLC‐OVA‐bearing CD45.2 mice (*n* = 5 biological replicates). (B) Representative images of tumors. (C, D) Tumor growth and survival curve of *Hs1bp3* ^+/+^ or *Hs1bp3* ^−/−^ CD8^+^ OT‐1 T cell‐treated mice. (E, F) Proportions and numbers of tumor‐infiltrating CD44^+^CD62L^+^ and CD44^+^CD62L^−^ subsets determined by flow cytometry on day 14. (G,H) Cytometric evaluation of Tcf‐1 level in CD8^+^ T cells from *Hs1bp3* ^+/+^ or *Hs1bp3* ^−/−^ models, along with secretion of IFN‐γ, Granzyme‐B and IL‐2. (I) Diagram of the rechallenge model. (J,K) Tumor volume and Kaplan‐Meier survival curves for CD45.2 mice after rechallenging with 1 × 10^6^ LLC‐OVA tumor cells (*n* = 5 biological replicates). (L,M) Proportions and numbers of CD44^+^ subsets in rechallenged mice. (N–O) The expression levels of Tcf‐1 and pro‐inflammatory mediators. Value is expressed as mean ± SEM. Statistical significance was evaluated by unpaired two‐tailed Student's t‐test (E–H, L–O). Two‐way ANOVA was performed to determine the significance of tumor growth (C,J), and survival curves were evaluated via the log‐rank Mantel‐Cox test (D, K). *, *P* < 0.05; **, *P* < 0.01; ***, *P* < 0.001; ****, *P* < 0.0001; ns: no significance.

### 2.4 HS1BP3 Ablation Impairs CD8^+^ T Cell Responses through Alterations in Nicotinamide Metabolism

To investigate the underlying mechanisms by which Hs1bp3 deficiency impairs antitumor responses and memory CD8^+^ T cell differentiation, we conducted comprehensive metabolomic profiling of sorted activated CD8^+^ T cells from *Hs1bp3* ^−/−^ and *Hs1bp3* ^+/+^ OT‐1 mice. Interestingly, pathway profiling analysis revealed pronounced upregulation of nicotinate and nicotinamide metabolism in Hs1bp3‐deficient CD8^+^ T cells (Figure 4A), with increased levels of NAD^+^ (Figure 4B), which is a key substrate of the deacetylase Sirt1. Hs1bp3 deficiency increased the levels and activity of Sirt1 (Figure 4C–E and Figure S7A). To determine whether HS1BP3 associates with SIRT1, co‐immunoprecipitation assays were performed using control and HS1BP3‐overexpressing Jurkat cells. HS1BP3 was specifically enriched in SIRT1 immunoprecipitates but not in the IgG control. This indicated that HS1BP3 associates with SIRT1 (Figure S7B). We further treated *Hs1bp3* ^−/−^ CD8^+^ T cells with a Sirt1 inhibitor in vitro, which resulted in a higher percentage of CD44^+^CD62L^+^ T cells (Figure S7C) and increased level of memory related genes compared with controls (Figure S7D). Meanwhile, Sirt1 shRNA substantially reduced Sirt1 expression (Figure S7E). shSirt1 cells showed a marked increase in the proportion of CD44^+^CD62L^+^ T cells (Figure S7F). A similar effect was observed in *Hs1bp3* ^−/−^ CD8^+^ T cells primed with FK866 in vitro, an inhibitor of nicotinamide phosphoribosyltransferase (NAMPT) (Figure S7G–J), which suggests that Sirt1 may mediate Hs1bp3‐regulated memory CD8^+^ T cell differentiation. To further assay the effect of Sirt1 on T cell memory fate in vivo, we utilized a model in which *Hs1bp3* ^−/−^ CD8^+^ T cells, subjected to in vitro inhibitor of Sirt1, were engrafted into CD45.2 mice following injection of *Lm*‐OVA. Sirt1 inhibitor supplementation led to the proliferation of CD44^+^CD62L^+/−^ T cells as well as Tcf‐1 expression (Figure 4F). In addition, treating Sirt1 inhibitor *Hs1bp3* ^−/−^ CD8^+^ T cells in vitro markedly attenuated in tumor volume and prolonged survival of mice (Figure 4G–I). The abundance of CD44^+^CD62L^+/−^ and Tcf‐1^+^ T cells were higher in the Sirt1 inhibitor group than in the control group, as well as the production of IFN‐γ (Figure 4J–L). Previous studies have reported that NAD^+^‐Sirt1‐CD38 axis orchestrated the processing of extracellular NAD^+^ to adenosine, thereby impairing the cytotoxic potency of CD8^+^ T cells in malignancy [19]. In this study, clinical assessment of CD8^+^ T cells isolated from patients with lung cancer demonstrated that SIRT1 activity is inversely linked to the secretion of cytokines, such as IFN‐γ, IL‐2 and Perforin (Figure 4M). These findings suggest that Hs1bp3 ablation impedes memory T cell differentiation and function through increased NAD^+^ levels and elevated Sirt1 activity.

**FIGURE 4:**
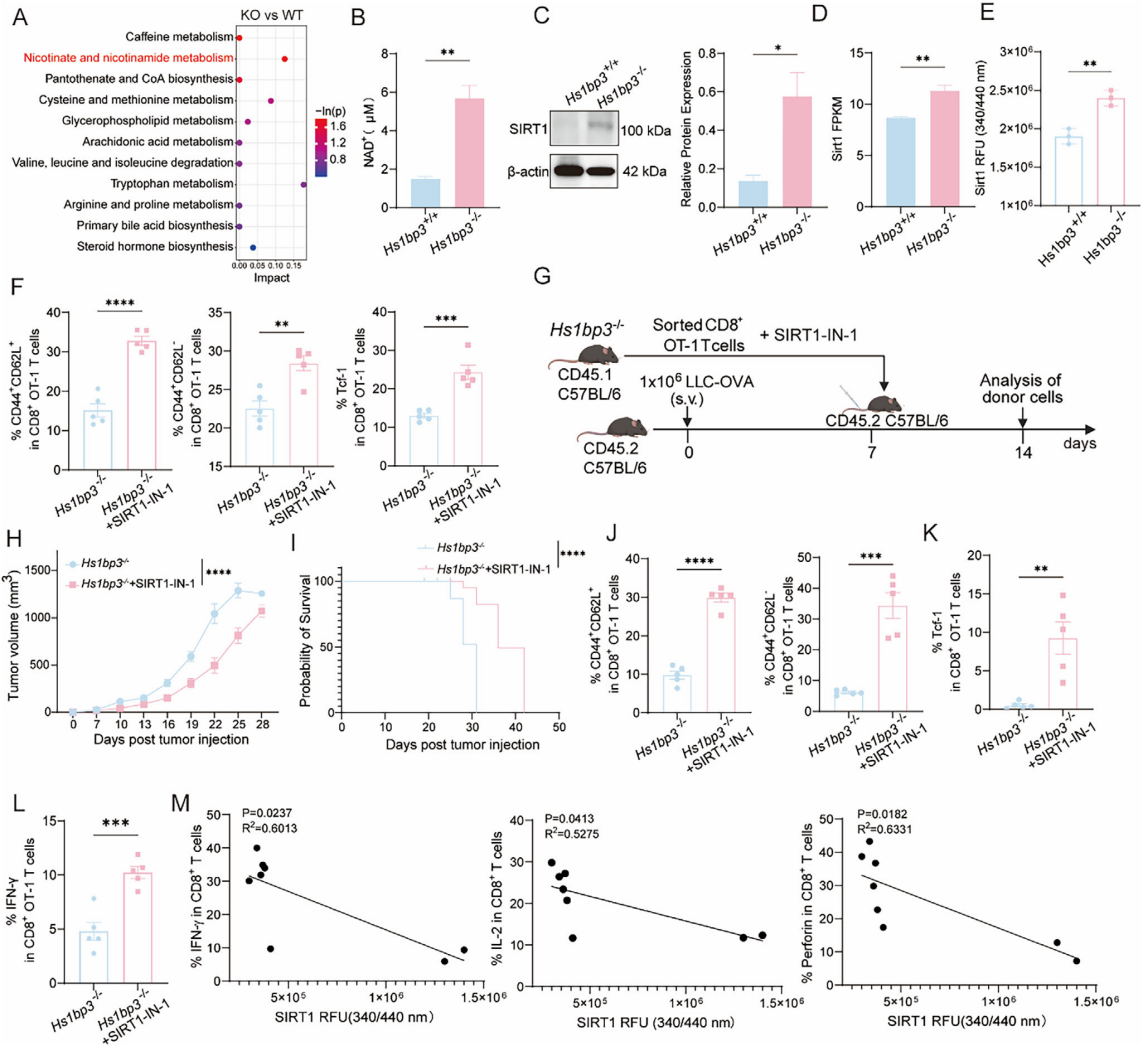
HS1BP3 deficiency impedes the differentiation of CD8^+^ T cells through activating NAD^+^‐sirt1 axis. (A) KEGG enrichment analysis of non‐targeted metabolomics analysis from *Hs1bp3* ^+/+^ or *Hs1bp3* ^−/−^ CD8^+^ OT‐1 T cells (*n* = 4 biological replicates). (B) The level of NAD^+^ was detected in *Hs1bp3* ^+/+^ or *Hs1bp3* ^−/−^ CD8^+^ OT‐1 T cells using an NAD^+^ /NADH assay Kit (*n* = 3 biological replicates). (C) Western blot of SIRT1 expression (*n* = 3 biological replicates). This experiment was independently performed three times. (D)FPKM of Sirt1 expression determined by RNA‐seq profiling of *Hs1bp3* ^+/+^ or *Hs1bp3* ^−/−^ CD8^+^ OT‐1 T cells (*n* = 4 biological replicates). (E) The activity of Sirt1 in CD8^+^ T cells (*n* = 3 biological replicates). (F) Flow cytometry frequencies of CD44^+^CD62L^+^, CD44^+^CD62L^−^ and Tcf‐1^+^ CD8^+^ T cells (*n* = 5 biological replicates). (G) Schematic diagram of Sirt1 inhibitor treated *Hs1bp3* ^−/−^ CD8^+^ OT‐1 T cells in tumor bearing mice. (H, I) Tumor progression and Kaplan‐Meier survival estimate of mice in *Hs1bp3* ^−/−^ or Sirt1 inhibitor primed *Hs1bp3* ^−/−^ CD8^+^ T cells (*n* = 5 biological replicates). (J) The proportions of CD44^+^CD62L^+^ and CD44^+^CD62L^−^ T cell subsets, (K) Tcf‐1^+^, and (L) IFN‐γ^+^ T cells in mouse tumor samples treated with *Hs1bp3* ^−/−^ or Sirt1 inhibitor primed *Hs1bp3* ^−/−^ CD8^+^ T cells (*n* = 5 biological replicates). (M) *Ex vivo* assessment of CD8^+^ T cells in PBMCs of patients with lung cancer (*n* = 8 patients). The SIRT1 activity and pro‐inflammatory cytokines were detected using ELISA or flow cytometry, respectively. Values are expressed as mean ± SEM. Statistical significance was evaluated by unpaired two‐tailed Student's t‐test (B–F and J–L). Two‐way ANOVA was performed to value the significance of tumor growth (H), and survival curves were analyzed using the log‐rank Mantel‐Cox test (I). *, *P* < 0.05; **, *P* < 0.01; ***, *P* < 0.001; ****, *P* < 0.0001.

Nicotinamide acts as a precursor of visfatin and nicotinamide N‐methyltransferase (NNMT), thereby driving the synthesis of NAD^+^ and 1‐Methylnicotinamide (MNAM), respectively [29]. Subsequently, MNAM was enriched in the Hs1bp3 ablation group (Figure 5A), which impaired the antitumor response in cancer [30]. Moreover, the MNAM activity in the supernatant of CD8^+^ T cells from *Hs1bp3* ^−/−^ OT‐1 mice was evaluated using an ELISA assay. MNAM levels were increased in *Hs1bp3* ^−/−^ CD8^+^ T cells (Figure 5B). After treating *Hs1bp3* ^+/+^ CD8^+^ T cells with MNAM, the levels of IFN‐γ, Granzyme B, and IL‐2 were significantly decreased (Figure 5C), whereas treatment with an inhibitor of MNAM generation in *Hs1bp3* ^−/−^CD8^+^ T cells reversed these effects (Figure 5D,E). To investigate whether MNAM suppressed the tumor‐killing potency of CD8^+^ T cells, *Hs1bp3* ^+/+^ CD8^+^ T cells, exposed to MNAM in vitro, were transferred into mice bearing LLC‐OVA tumors. MNAM treatment substantially accelerated tumor progression and shortened the survival (Figure 5F, H). The proportions of CD44^+^CD62L^+/−^ T cells had no significant difference between the two groups (Figure 5I). However, intratumoral CD8^+^ T cells displayed lower levels of activation‐associated markers (CD28 and CD69), and higher levels of PD‐1 than those of their control counterparts (Figure 5J). Additionally, MNAM supplementation suppressed intracellular cytokine production in CD8^+^ T cells (Figure 5K), indicating impaired tumor‐killing capability of CD8^+^ T cells under Hs1bp3 deficiency.

**FIGURE 5:**
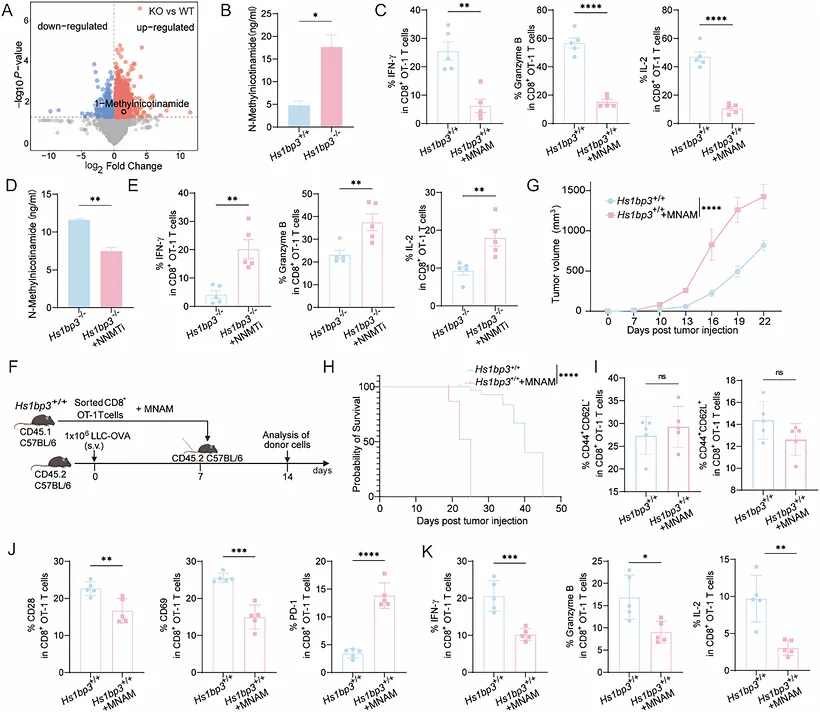
HS1BP3 deficiency leads to functional impairment in CD8^+^ T cells through NAM‐dependent accumulation of MNAM. (A) Volcano plot showing metabolites among *Hs1bp3* ^+/+^ and *Hs1bp3* ^−/−^ CD8^+^ OT‐1 T cells (*n* = 4 biological replicates). (B) The MNAM levels of *Hs1bp3* ^+/+^ and *Hs1bp3* ^−/−^ CD8^+^ T cells were detected using ELISA (*n* = 3 biological replicates). (C) The secretory repertoire of IFN‐γ, Granzyme‐B and IL‐2 in *Hs1bp3* ^+/+^ CD8^+^ OT‐1T cells treated with or without MNAM (*n* = 5 biological replicates). (D‐E) The MNAM levels and pro‐inflammatory cytokines were detected in *Hs1bp3* ^−/−^ CD8^+^ OT‐1 T cells treated with or without NNMTi (*n* = 3 biological replicates). (F) Schematic diagram of the *Hs1bp3* ^+/+^ CD8^+^ OT‐1 T cells treated with or without MNAM in LLC‐OVA tumor bearing hosts. (G‐H) Tumor progression and survival curves of LLC‐OVA tumors in CD45.2 C57BL/6 mouse (*n* = 5 biological replicates). (I) The proportions of CD44^+^CD62L^+^ and CD44^+^CD62L^−^ T cell subsets in MNAM treatment group and control group (*n* = 5 biological replicates). (J) Analysis of CD28, CD69 and PD‐1 abundance in *Hs1bp3* ^+/+^ CD8^+^ OT‐1 T cells treated with or without MNAM within tumors at day 14 (*n* = 5 biological replicates). (K) Percentages of IFN‐γ^+^, Granzyme‐B^+^ and IL‐2^+^ T cells in tumor samples from mice treated with *Hs1bp3* ^+/+^ or MNAM primed *Hs1bp3* ^+/+^ CD8^+^ OT‐1 cells (*n* = 5 biological replicates). Data are expressed as mean ± SEM. Statistical significance was evaluated by unpaired two‐tailed Student's t‐test (B–E, I–K). Two‐way ANOVA was showed to figure out the significance of tumor volume (G), and survival curves were analyzed using the log‐rank Mantel‐Cox test(H). *, *P* < 0.05; **, *P* < 0.01; ***, *P* < 0.001; ****, *P* < 0.0001; ns: no significance.

### 2.5 SIRT1 Activation Upon HS1BP3 Deletion Impedes Memory T Cell Differentiation through the FOXO3‐BCL6 Pathway

SIRT1 regulates diverse biological processes and hampers T‐cell responses through multiple transcription factors, including FOXO proteins. Therefore, we hypothesized that Hs1bp3 is a negative regulator of Sirt1, and dysregulation of Hs1bp3 may disrupt Foxo3 [31]. To investigate this hypothesis, transcriptomic profiling of *Hs1bp3* ^−/−^ and *Hs1bp3* ^+/+^ CD8^+^ T cells were conducted. KEGG and GSEA analysis indicated that Foxo pathway was enriched in *Hs1bp3* ^−/−^ CD8^+^ T cells (Figure 6A,B). Consistent with these findings, Foxo3 levels were elevated in *Hs1bp3* ^−/−^ CD8^+^ T cells and co‐localized with Sirt1 expression (Figure 6C,D). Furthermore, HS1BP3 overexpression inhibited SIRT1 expression, and increased FOXO3 acetylation, supporting that increased FOXO3 acetylation was associated with reduced FOXO3 protein abundance in the Jurkat cell model (Figure S8A). Meanwhile, CD8^+^ T_eff_ cells, rather than T_m_ cells, exhibited high expression of FOXO3 and SIRT1 (Figure S8B). Notably, FK866 treatment decreased FOXO3 expression (Figure 6E), whereas Foxo3 knockdown (Figure S8C) did not alter NAD^+^ levels or SIRT1 abundance in *Hs1bp3* ^−/−^ CD8^+^ T cells (Figure 6F,G). Collectively, these results collectively position Foxo3 as a downstream regulator of the Sirt1‐mediated pathway. Foxo3 restrains T cell function by disturbing the fate of memory during viral infection [31]. In this study, we observed that the CD44^+^CD62L^+^ subset, memory‐related gene expression levels and mitochondrial OXPHOS proteins were markedly increased in Foxo3 down‐regulating group (Figure S8D–F). To investigate the mechanism by which HS1BP3 deficiency reconfigures Foxo3‐mediated transcription, we integrated CUT&Tag to pinpoint target genes of Foxo3 in *Hs1bp3* ^−/−^ and *Hs1bp3* ^+/+^ CD8^+^ T cells (Figure S8G,H), and KEGG pathway analysis was used to elucidate epigenetic regulatory landscape (Figure S8I). Foxo3 was enriched in the Bcl6 promoter in the Hs1bp3 ablation group (Figure 6H). Bcl6 is a critical transcription factor involved in chromatin remodeling of T_m_‐specific genes [32]. Motif enrichment analysis of Foxo3 demonstrated the concurrent enrichment of Bcl6 within its binding region (Figure 6I). ChIP‐qPCR analysis revealed that the anti‐Foxo3 antibody specifically enriched the Bcl6 promoter region (Figure 6J). Moreover, the expression of BCL6 was decreased in the Hs1bp3 deletion group (Figure 6K). To validate the functional impact of this binding, a dual‐luciferase reporter assay showed that mutant (MUT) Bcl6 promoter decreased luciferase activity, thus confirming that Foxo3 inhibited the expression of Bcl6 (Figure 6L,M). Foxo3 knockdown eliminated the effects of Sirt1 activation on Bcl6 expression (Figure S8J). Additionally, Bcl6 expression was substantially increased in the Bcl6‐overexpression group (Figure S8K), whereas Sirt1 and Foxo3 expression did not differ between the control and Bcl6‐overexpression groups (Figure S8L,M). This suggested that Foxo3 serves as a critical node for the Sirt1‐mediated regulation of Bcl6 in CD8^+^ T cells. Interestingly, Bcl6 overexpression increased the proportion of CD44^+^CD62L^+^ OT‐1 T cells (Figure S8N). Thus, these results indicate that Hs1bp3 ablation activates the Sirt1‐Foxo3 axis and leads to the epigenetic repression of Bcl6. This underscores the key determinant of Hs1bp3 in orchestrating memory CD8^+^ T cell formation.

**FIGURE 6:**
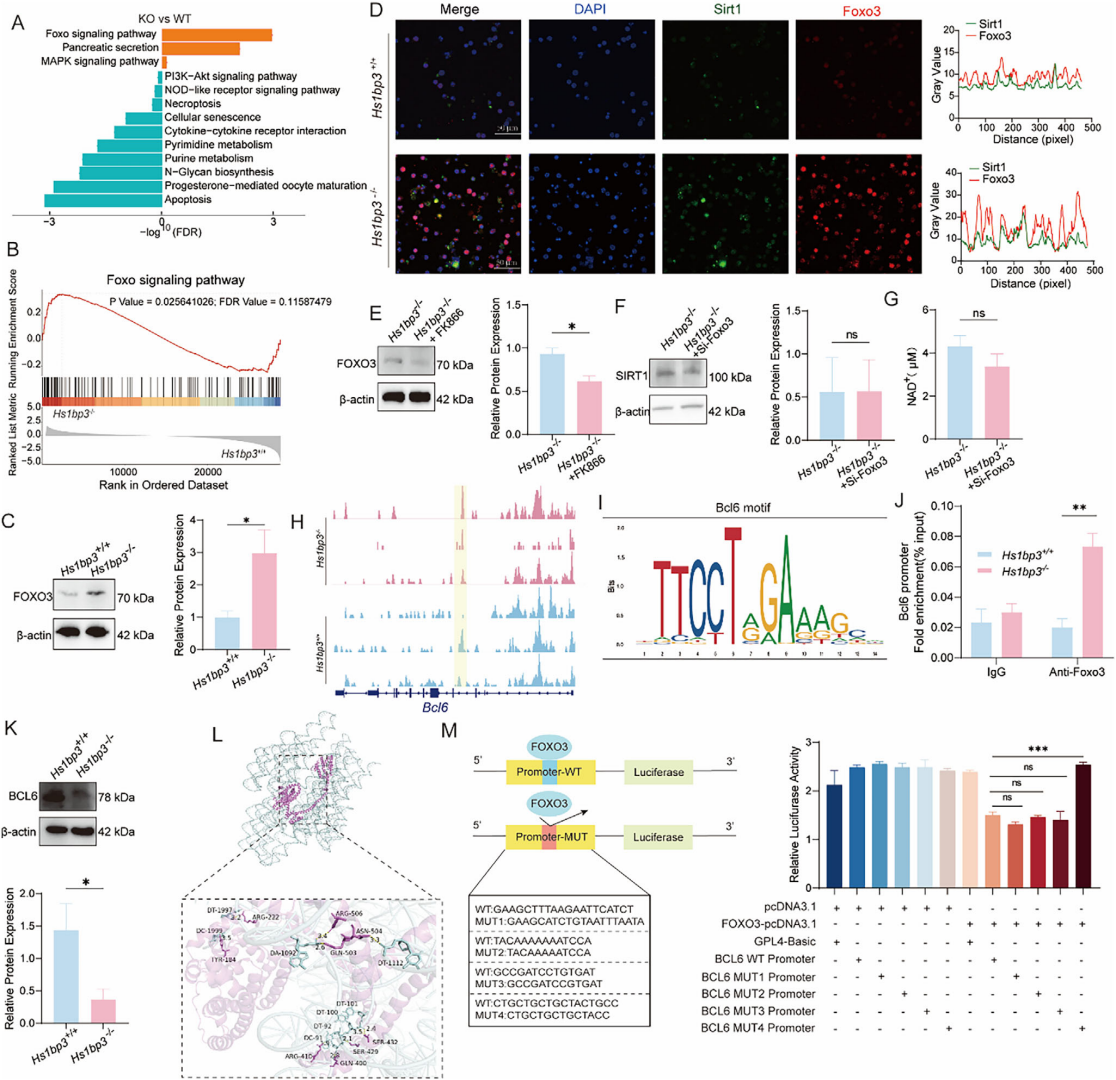
SIRT1‐FOXO3 axis suppresses BCL6 activity in HS1BP3‐deficient CD8^+^ T cells. (A‐B) RNA was from *Hs1bp3* ^+/+^ and *Hs1bp3* ^−/−^ CD8^+^ OT‐1 cells for transcriptome analysis (*n* = 4 biological replicates). In *Hs1bp3* ^−/−^ CD8^+^ OT‐1 cells, KEGG and GSEA analysis displayed a prominent enrichment signature in the Foxo signaling pathway. (C) Western blot analysis of FOXO3 expression levels in *Hs1bp3* ^+/+^ and *Hs1bp3* ^−/−^ CD8^+^ OT‐1 T cells (*n* = 3 biological replicates). This experiment was independently performed three times. (D) Illustrative micrographs of immunofluorescence staining of Sirt1 (green) and Foxo3 (red) in *Hs1bp3* ^+/+^ and *Hs1bp3* ^−/−^ CD8^+^ OT‐1 T cells, Scale bar  =  50 µm (*n* = 3 biological replicates). (E, F) *Hs1bp3* ^−/−^ CD8^+^ OT‐1 cells were treated with 0.3 nM FK866 or transfected with Foxo3 siRNA. The abundance of protein FOXO3 and SIRT1 were characterized via western blotting (*n* = 3 biological replicates). This experiment was independently performed three times. (G) The NAD^+^ levels of *Hs1bp3* ^−/−^ CD8^+^ OT‐1 cells following transfection with Foxo3 siRNA were observed via NAD^+^ /NADH assay Kit (*n* = 3 biological replicates). (H) IGV tracks for Bcl6 from Foxo3 CUT&Tag analysis, showing that Foxo3 is enriched in the Bcl6 promoter region. (*n* = 3 biological replicates). (I) Predicted binding sites of Foxo3 on the Bcl6 promoter. (J) ChIP‐qPCR was used to detect the Bcl6 promoter enrichment of Foxo3 and IgG in the target DNA region, cells were from *Hs1bp3* ^+/+^ and *Hs1bp3* ^−/−^ CD8^+^ OT‐1 T cells. This experiment was independently performed three times. (K) Protein levels of BCL6 in *Hs1bp3* ^−/−^ CD8^+^ OT‐1 T cells were determined via western blotting (*n* = 3 biological replicates). This experiment was independently performed three times. (L) The main interactions between Foxo3 and Bcl6 are shown. (M) Luciferase intensity of Jurkat cells transfected with the BCL6 reporters (*n* = 3 biological replicates). Data are expressed as mean ± SEM. Statistical significance was evaluated by unpaired two‐tailed Student's t‐test (C, E–G, J, K) and one‐way ANOVA (M). *, *P* < 0.05; **, *P* < 0.01; ***, *P* < 0.001; ns: no significance.

### 2.6 CAR‐T Cells Co‐Expressing HS1BP3 Exhibit a Memory Phenotype

Next, the effects of HS1BP3 on the promotion of the CAR‐T cells memory‐like program were investigated. Lentiviral vectors simultaneously expressing Meso‐CAR and HS1BP3 were constructed (Figure 7A), and Meso‐HS1BP3 CAR‐T cells were produced from the peripheral blood mononuclear cell (PBMC) samples (Figure 7B,C). Cytokine release and tumor lysis analyses showed that HS1BP3 overexpressed CAR‐T cells increased IFN‐γ, Granzyme‐B and Perforin secretion as well as tumor lysis capability compared to Meso‐CAR‐T cells upon co‐incubation with H322 tumor cells (Figure 7D,E). Furthermore, we performed a repeated stimulation assay in which Meso‐CAR‐T cells were counted and re‐challenged with fresh H322 every two days at an effector‐to‐target (E:T) ratio of 5:1 throughout the experiment, as previously reported [33]. The Meso‐HS1BP3 CAR‐T cells exhibited significantly higher expansion capacity and enhanced antitumor activity than control CAR‐T cells (Figure S9A,B). Furthermore, HS1BP3 overexpression increased the proportion of CD45RA^−^CD62L^+^ (T_cm_), and upregulated mitochondrial OXPHOS proteins compared to those in control CAR‐T cells (Figure S9C and Figure 7F,G). Meso‐HS1BP3 CAR‐T cells displayed higher basal and maximal respiration rates and spare respiratory capacity, thereby indicating enhanced oxidative phosphorylation (Figure 7H). Mechanistically, Meso‐HS1BP3 CAR‐T cells showed a diminished abundance of SIRT1 (Figure 7I, Figure S9D) and MNAM compared with that in controls (Figure 7J). Bioluminescence imaging (BLI) assays revealed that tumor burden was increased in MNAM‐treated Meso‐HS1BP3 CAR‐T cells (Figure 7K). The HS1BP3 expression also decreased FOXO3 levels and increased BCL6 levels in CAR‐T cells (Figure 7L,M, Figure S9E,F). Collectively, these results indicated that CAR‐T cells expressing HS1BP3 are characterized by memory properties and prolonged cytolytic activity upon antigen exposure.

**FIGURE 7:**
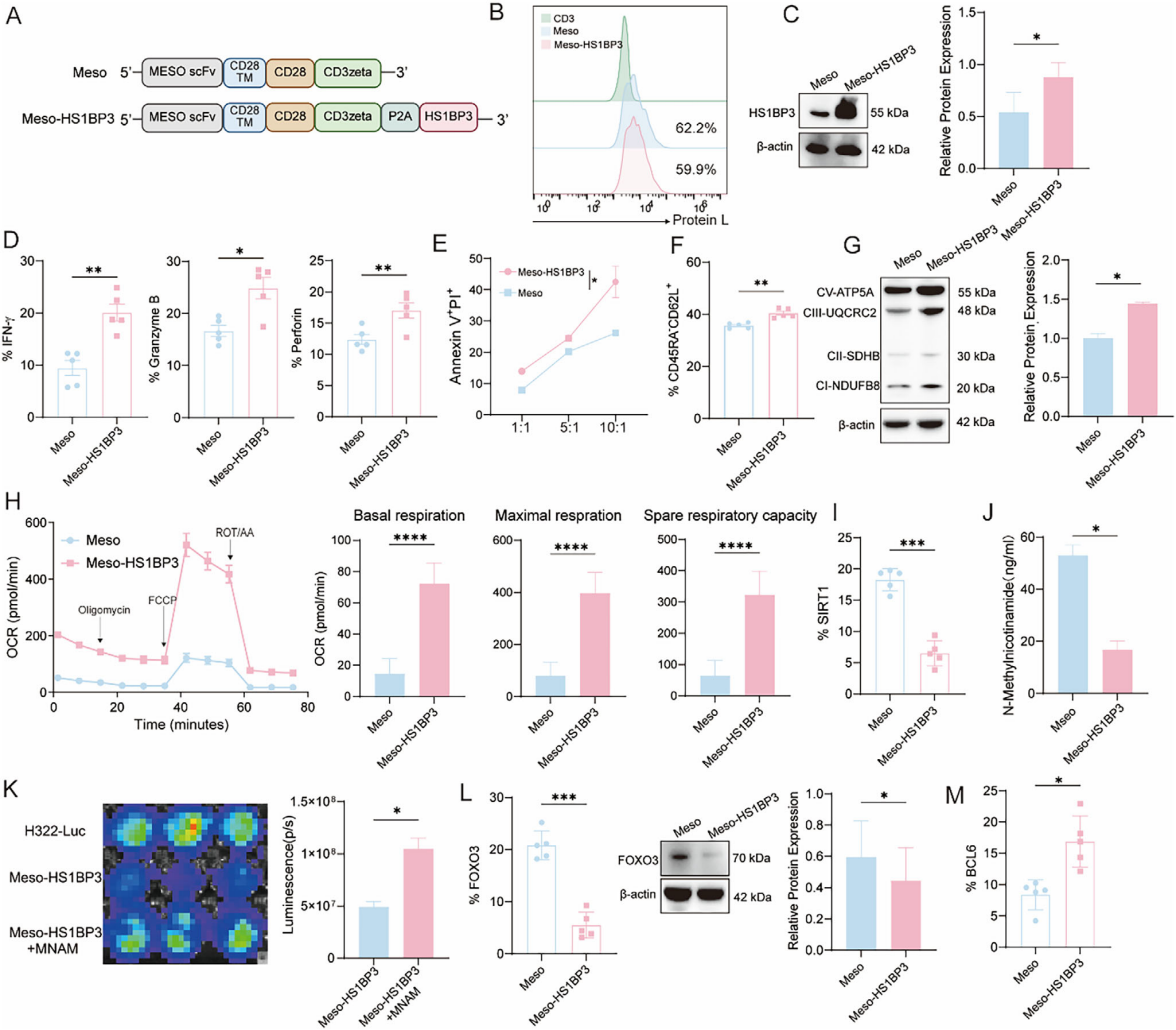
HS1BP3 overexpression enhances memory differentiation of CAR‐T cells. (A) CARs’ Structures. (B) Representative flow plot showing Protein L levels on CD3^+^ T cells. (C) The expression of HS1BP3 in CAR‐T cells (*n* = 3 healthy donors). This experiment was independently performed three times. (D) The secretions of IFN‐γ, Granzyme‐B, and perforin in CAR‐T cells afterco ‐ culture with H322 cells (*n* = 5 healthy donors). (E) Specific lysis of H322 cells after exposure to CAR‐T cells, E:T ratios are listed (*n* = 5 healthy donors). (F) Percentage of T_cm_ (CD45RA^−^CD62L^+^) CAR‐T cells. (G) Expression levels of mitochondrion OXPHOS proteins (*n* = 3 healthy donors). This experiment was independently performed three times. (H) The OCRs of CAR‐T cells were detected via seahorse energy detection (*n* = 5 healthy donors). (I) Analysis of SIRT1 in CAR‐T cells through flow cytometry (*n* = 5 healthy donors). (J) The MNAM level of CAR‐T cells was measured by ELISA (*n* = 3 healthy donors). (K) Cytotoxicity of CAR‐T against luc‐H322 (24 h, co‐culture) under MNAM‐induced metabolic stress conditions. BLI assays determined tumor cell viability (*n* = 3 healthy donors). (L,M) Intracellular titers of FOXO3 and BCL6 in CAR‐T cells accessed by flow cytometry (*n* = 5 healthy donors) and western blotting (*n* = 3 healthy donors). This experiment was independently performed three times. Values are expressed as mean ± SEM. Statistical significance was evaluated by paired two‐tailed Student's t‐test (C, D, F–M) and two‐way ANOVA (E). *, *P* < 0.05; **, *P* < 0.01; ***,*P < 0.001;* ****, *P* < 0.0001.

### 2.7 HS1BP3 Overexpression Enhances CAR‐T Cell Function in Solid Tumors

Luciferase‐H322 cells were engrafted into NOD‐SCID mice, and the efficacy of CAR‐T cells was assessed in a preclinical xenograft model. Compared to mock and Meso‐CAR‐T cells, group treated with Meso‐HS1BP3 CAR‐T cells showed accelerated tumor debulking and enhanced overall survival (OS) (Figure 8A–C). Moreover, these cells demonstrated a robust expansion capacity (Figure 8D), and inhibited the expression of exhaustion markers (Figure 8E). Consistent with their function in the tumor microenvironment, HS1BP3‐transduced CAR‐TILs showed significantly increased frequencies of CD45RA^−^CD62L^+^ (Figure 8F). The level of IFN‐γ, perforin and Granzyme‐B were higher in tumor infiltrating Meso‐HS1BP3 CAR‐T cells than in control cells (Figure 8G). To ensure the clinical translational potential of this approach, it was imperative to ascertain whether HS1BP3‐overexpressing CAR‐T cells elicit systemic toxicities or adverse pathologies in non‐tumorigenic tissues. Consequently, a comprehensive safety evaluation was conducted. The histopathological examination of the major organs revealed no overt treatment‐related abnormalities in mice treated with Meso‐HS1BP3 CAR‐T cells (Figure 8H).

**FIGURE 8:**
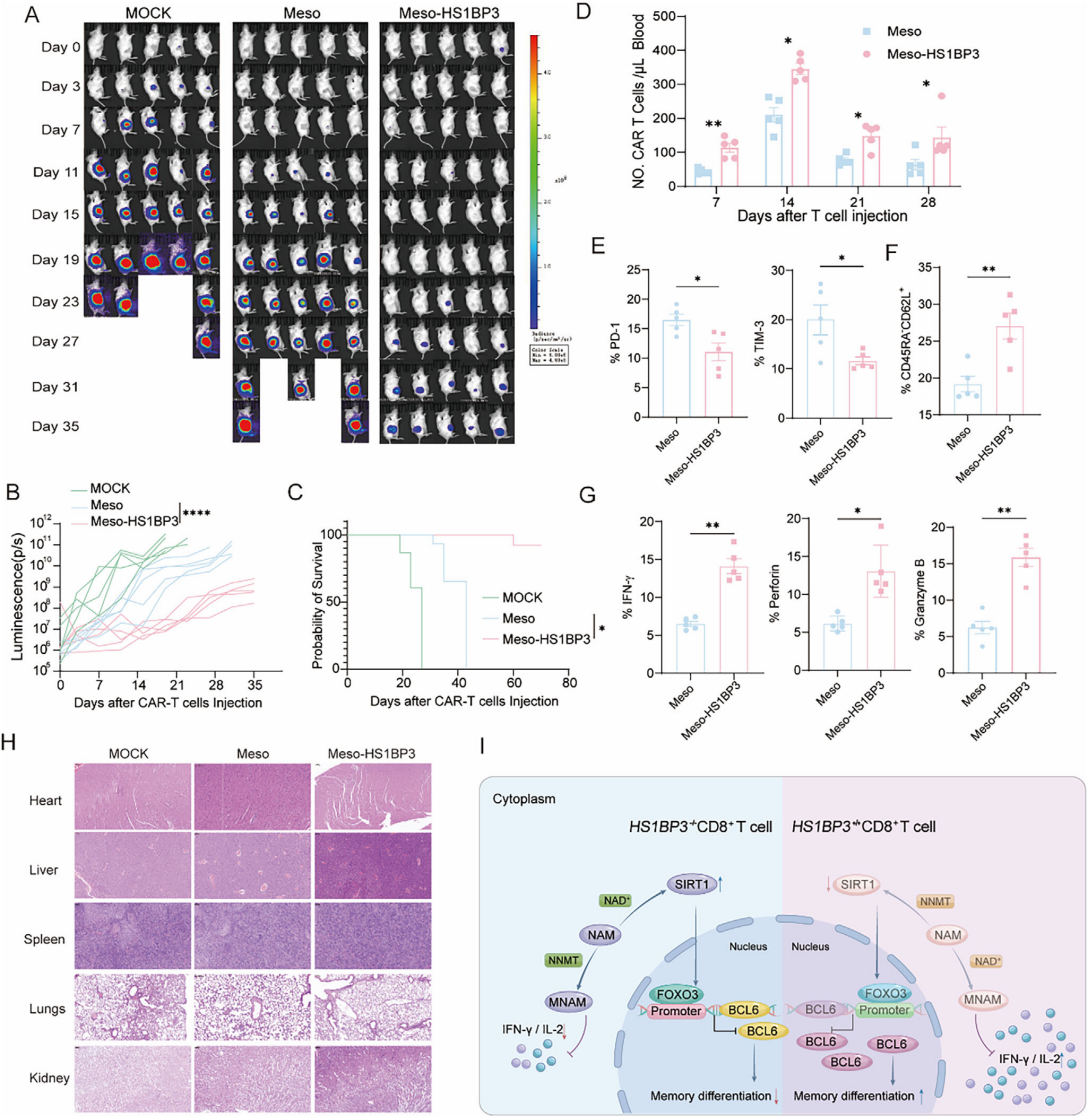
HS1BP3 overexpression enhances the efficacy of CAR‐T cells in a mouse tumor transplant model. (A) BLI assay of NOD‐SCID mice challenged with 2 × 10^5^ luci‐H322 tumor cells, and 1 × 10^6^ Meso or Meso‐HS1BP3 CAR T cells (n = 5 biological replicates). (B) Quantitative assessment of the bioluminescence of mice receiving treatment cohorts. (C) Survival curve of mice in different groups. (D) The numbers of CAR‐T cells in blood of mice at specific time points. (E) PD‐1^+^ and TIM‐3^+^ CAR‐T cells, (F) Proportions of T_cm_ (CD45RA^−^CD62L^+^) in tumor tissues analyzed via flow cytometry. (G) Expression of cytokines (IFN‐γ, Perforin, and Granzyme B) in tumor‐infiltrating CAR‐T cells. (H) Hematoxylin and eosin(H&E) histology staining outcomes for heart, liver, spleen, lungs and kidney of mice on day 7 post treatment. Scale bar = 50 µm. (I) Schematic diagram of HS1BP3 orchestrating memory CD8^+^ T cell Fate determination. Values are expressed as mean ± SEM. Evaluating Statistical significance by paired two‐tailed Student's t‐test (D‐G). Two‐way ANOVA was performed to assess the significance of tumor volume (B), and survival curves were analyzed using the log‐rank Mantel‐Cox test (C). *, *P* < 0.05; **, *P* < 0.01; ***, *P* < 0.001.

In summary, HS1BP3 enhanced T cell function by concurrently inhibiting the MNAM, and suppressing the SIRT1‐FOXO3 signaling axis to upregulate BCL6 activity, thereby promoting T_m_ cell differentiation (Figure 8I). Thus, HS1BP3 overexpression could be a promising strategy to optimize CAR‐T cell therapeutic durability for solid tumors.

## 3 Discussion

Promoting commitment to long‐lived Tm cells, which retain plasticity and robust recall capacity, represents a promising strategy for improving immunotherapy [34, 35, 36]. Despite its reported involvement in T cell immune regulation, the role of Hs1bp3 in memory T cell differentiation remains largely elusive. In this study, we found that HS1BP3 as a potential regulator of memory CD8^+^ T cell differentiation, possibly through its association with metabolic and transcriptional programs. To dissect the role of Hs1bp3 in T cell differentiation, a *Lm*‐OVA challenge model was utilized to generate foreign antigen (OVA)‐specific memory OT‐1 T cells. This system allowed us to closely mimic memory T cells formed by natural infection [3]. Hs1bp3 deficiency markedly impaired memory CD8^+^ T cell development, which was also linked to mitochondrial dysfunction, including reduced mitochondrial OXPHOS proteins and OCR. In a previous study, memory‐related genes such as *Il‐7r* [37]*, Lef‐1* [38], *Tcf‐7* [39], *Sell* and *Bcl6* [11] were markedly decreased in Hs1bp3‐deficent CD8^+^ T cells, which exhibited impaired recall responses in vivo. Given the superior tumor‐controlling capacity of T_m_ cells [40, 41], the current study employed an adoptive transfer model to demonstrate that Hs1bp3 deficiency limits the accretion of tumor‐infiltrating T_m_ cells, thereby compromising antitumor immunity. These findings indicate that Hs1bp3 is a key regulator of memory T cell fate determination and function.

Cellular metabolism is recognized as an important factor in determining memory T cell fate. Consistent with this concept, our results showed that Hs1bp3 ablation increased intracellular NAD^+^ levels. As a fundamental biological element, NAD^+^ has a profound influence on metabolism, which is intricately coupled with the tumor‐killing functionality of T cells [42, 43]. However, NAD^+^ serves as a substrate for various NAD^+^‐consuming enzymes such as Sirt1 [44], which deacetylates Foxo3a, and suppresses immune response. Foxo3 has been implicated in inhibiting anti‐viral T cell function via constraining the establishment of enduring memory compartments. This phenomenon has been observed in both HIV patients and murine models of LCMV infection [45, 46]. The current study further demonstrated that Hs1bp3‐deficient CD8^+^ T cells had increased NAD^+^ availability and sustained Sirt1 activity. Meanwhile, the inhibitor of Sirt1 treated *Hs1bp3^−/−^* CD8^+^ T cells restored T_m_ cell formation and the anti‐tumor response, suggesting that NAD^+^‐Sirt1 axis regulates Hs1bp3 mediated T_m_ cell differentiation. Additionally, deletion of Hs1bp3 resulted in the enrichment of the Foxo signaling pathway, accompanied by an accumulation of Foxo3 in CD8^+^ T cells. Importantly, Foxo3 and Bcl6 share overlapping binding regions, as verified by CUT&Tag‐seq and dual‐luciferase reporter assays. Bcl6 is recognized as a memory‐related gene; activation of the Sirt1‐Foxo3 axis suppresses memory T cell differentiation by inhibiting Bcl6 expression.

MNAM is a product of nicotinamide methylation, which can limit the antitumor immune response in ovarian cancer. Furthermore, MNAM is enriched in tumor‐ infiltrating CD8^+^ T cells, which suppressed T cells to elaborate the tumor‐promoting cytokine tumor necrosis factor alpha [30]. To further determine whether Hs1bp3 ablation regulates MNAM levels via NAD^+^ metabolism, metabolomic analyses were performed. The Hs1bp3‐deficient T cells exhibited increased MNAM levels, and treatment of CD8^+^ T cells with MNAM promoted tumor growth. Additionally, the MNAM reduced IFN‐γ, Granzyme‐B and IL‐2 levels in tumor—infiltrating CD8^+^ T cells. These findings support the conclusion that Hs1bp3 deficiency suppresses the tumor‐killing function of CD8^+^ T cells via MNAM accumulation.

CAR‐T cell therapy is a potent modality for treating malignancies in patients whose responses are intrinsically linked to a less‐differentiated phenotypic signature and the capacity to accomplish durable engraftment [47, 48, 49]. Accordingly, the current study tested the hypothesis that the genetic reinforcement of HS1BP3 could remodel CAR‐T cells to durably exist and sustain their antitumor activity. Our results showed that HS1BP3 enhanced memory formation of CAR‐T cells and their antitumor function in vivo. The survival of mice in the HS1BP3‐CAR‐T cell treatment group was prolonged, providing a promising strategy for cancer therapy.

In conclusion, this study identifies Hs1bp3 as an important regulator that promotes memory CD8^+^ T cell differentiation through modulation of the SIRT1–FOXO3‐BCL6 axis. Moreover, the MNAM accumulation associated with HS1BP3 deficiency contribute to impaired antitumor function of CD8^+^ T cells. Notably, HS1BP3 overexpression significantly delayed tumor progression in solid tumor models and increased CAR‐T cells persistence. Therefore, these results serve as a firm foundation of effective CAR‐T therapies for solid malignancies through HS1BP3 overexpression.

## 4 Methods

### 4.1 Human Samples

The human peripheral blood samples and patients with lung cancer were obtained following approval from the Ethics Committee First Affiliated Hospital of Zhengzhou University (2025‐KY‐1023) and informed consent was obtained fromall participants. The clinical characteristics of patients are summarized in Table S1. The study complies with all the relevant ethical guidelines for research involving human participants.

### 4.2 Mice

*Hs1bp3^+/+^* and *Hs1bp3^−/−^* CD45.1^+^OT‐1 mice with a C57BL/6J background were generated by Cyagen Bioscience Inc. (GuangZhou, China). *Hs1bp3^+/+^* and *Hs1bp3^−/−^* CD45.1^+^OT‐1 mice were obtained through crossing *Hs1bp3^+/−^* CD45.1^+^ OT‐1 mice. Wild‐type CD45.2 C57BL/6J mice and NOD‐SCID mice were obtained from SPF Biotechnology Co. (Beijing, China). *Hs1bp3^+/+^* and *Hs1bp3^−/−^* CD45.2^+^ mice were a gift from Dr. Shengdian Wang. All experiments used mice aged 7–8 weeks. All animals grow in specific pathogen‐free (SPF) environment, and performed following institutional guidelines. The study received approval from the Animal Care and Use Committee of Zhengzhou University (ZZU‐LAC20210702[07]).

### 4.3 Cell Culture

H322‐luciferase, HEK293T, LLC, and LLC‐OVA cells were cultured in DMEM medium (Gibco, C11995500BT) enriched 10% fetal bovine serum (FBS; Biological Industries), 100 U mL^−1^ penicillin (Gibco, 15140‐122), and 100 µg mL^−1^ streptomycin (Gibco, 15140‐122). Cell lines mentioned were maintained at 37°C with 5% CO_2_.

### 4.4 Murine T Cell Isolation

Primary murine CD8^+^ T cells and CD45.1^+^ T cells were isolated from the spleens Using Mouse CD8^+^ T‐cell Isolation Kit (BioLegend, 480035) and Mouse CD45.1^+^ T‐cell Isolation Kit (BioLegend, 480118), respectively, following the manufacturer's instructions. Purified T cells grown with RPMI 1640 medium (Gibco,) enriched 10% FBS (FBS; Biological Industries), 50 IU mL^−1^ IL‐2 (Gibco, 212‐12‐5UG), 100 U mL^−1^ penicillin (Gibco, 15140‐122), and 100 µg mL^−1^ streptomycin (Gibco, 15140‐122). siRNA was designed and constructed by GenePharma (Shanghai, China).

The si‐Foxo3 specific sequences were as follows:

5′‐CAUGCGCGUUCAGAAUGAATT‐3′; 5′‐UUCAUUCUGAACGCGCAUGTT‐3′. Using Lipofectamine 3000 reagent (Invitrogen, L3000015) to transfect T cells.

### 4.5 *Lm*‐ova Immunization and CD8^+^ OT‐1 Cell Transfer

For adoptive T cell transfer, 1×10^5^ CD45.1^+^ CD8^+^ OT‐1 naïve T cells from 6–8 weeks old OT‐1 mice were delivered via tail vein injection into age‐matched CD45.2^+^ C57BL/6 recipient mouse. Recipient mice were injected *Lm*‐OVA (5×10^5^ c.f.u.) one day post‐transfer, and CD45.1^+^ T cells were analyzed and sorted for following experiments. *Listeria monocytogenes* expressing‐OVA (*Lm*‐OVA) was obtained from Dr. Bo Huang's laboratory.

*Hs1bp3^−/−^* and *Hs1bp3^+/+^* CD8^+^ OT‐1 T cells (1×10^5^) were transferred to CD45.2^+^ C57BL/6 mice. The recipients were intraperitoneally challenged with *Lm*‐OVA (5×10^5^ c.f.u.) on the following day. Splenocytes were harvested on day 30 after infection, and CD45.1^+^ T cells were subjected to flow cytometry.

To determine the recall response, CD8^+^ CD45.1^+^ T_cm_ cells were sorted from primary recipients at 30–35 days after initial infection and subsequently transferred into another wild‐type C57BL/6 mice (1×10^5^ cells per recipient). Recipient mice were intraperitoneally challenged with *Lm*–OVA the following day, and CD45.1^+^ T cells were assessed 13 days after the secondary infection.

To explore the function of Sirt1 in T cells differentiation, *Hs1bp3^−/−^* OT‐1 CD45.1^+^ CD8^+^ T cells (1×10^5^) were treated with 0.2 µM SIRT1‐IN‐1 (MedChemExpress, HY‐136199) for 48 h before adoptive transfer into WT C57BL/6 recipients. Hosts were infected by *Lm*–OVA (5×10^5^ c.f.u.) one day later. Splenic CD45.1^+^ T cells were assessed via flow cytometry on day 30 after infection.

### 4.6 Human T Cell Isolation

Peripheral blood mononuclear cells (PBMCs) were obtained using Human Peripheral Blood Lymphocyte Separation Medium (Beyotime, C0025). Total CD8^+^ or CD3^+^ T cells were sorted from PBMCs via Human CD8^+^ / CD3^+^ T Cell isolation kit (Miltenyi Biotec, 130‐096‐495, 130‐097‐043). Purified CD8^+^ T cells were stimulated by T Cell Activation/Expansion Kit (Miltenyi Biotec, 130‐091‐441) and IL‐2 (Gibco) for 48 h. To induce effector and memory T cell in vitro, cells were co‐cultured with IL‐2 or IL‐15 (10 ng mL^−1^, Gibco) for an additional 96 h.

### 4.7 qRT‐PCR

Total RNA was extracted via TRIzol reagent (Invitrogen, 15596018), followed by reverse transcription into cDNA with the Prime Script RT kit (TAKARA, RR037A). We performed quantitative PCR on a Bio‐Rad CFX96 platform using the primers listed: *Foxo3* (F: 5’‐GGGGAACCTGTCCTATGCC‐3’; R: 5’‐TTAGAGACCAAGAACTGGACGA‐3’); *Lef1* (F: 5’‐TCTGGCTACATAATGATGCCCA‐3’; R: 5’‐GGACATGCCTTGCTTGGAGTT‐3’); *Sell* (F: 5’‐TACATTGCCCAAAAGCCCTTAT‐3’; R: 5’‐CCTCCTTGGACTTCTTGTTGTT‐3’); *Tcf7* (F: 5’‐AACTGGCCCGCAAGGAAAG‐3’; R: 5’‐CTCCGGGTAAGTACCGAATGC‐3’); *Bcl6* (F: 5’‐CCTGCACTTAAACCTCCCCG‐3’; R: 5’‐GACCTCGGTAGGCCATGATG‐3’); *Il‐7r* (F: 5’‐AAAGTCCGATCCATTCCCCAT‐3’; R: 5’‐CCATCCTCCTTGATTCTTGGGT‐3’); *Gapdh* (F: 5’‐CTTCTTGTGCAGTGCCAGC‐3’; R: 5’‐GAGGTCAATGAAGGGGTCGT‐3’).

### 4.8 Western Blotting

Protein lysates were prepared by treating PBS‐washed cells with RIPA buffer (Thermo Fisher, 89901) containing a comprehensive inhibitor cocktail for proteases and phosphatases. Following a 10‐min centrifugation at 10,000 ×g (4°C), the protein‐rich supernatant was resolved on SDS‐PAGE gels and electro‐blottedonto nitrocellulose membranes. Using with 5% non‐fat milk block for membranes 1 hour at room temperature before being probed overnight with primary antibodies: anti‐Hs1bp3 (Proteintch); anti‐Sirt1; anti‐OXPHOS (Abcam); anti‐Foxo3a; anti‐β‐Actin (Cell Signaling Technology). Signals were developed via HRP‐linked secondary antibodies and captured using an ECL imaging system (Servicebio, G2020) at BLT GelView 6000 Plus system. Band intensities were quantified using ImageJ software.

### 4.9 Flow Cytometry

Cells were incubated with antibodies against surface markers for 15 min at 4°C. For intracellular cytokine staining, cells were stimulated with phorbol 12‐myristate 13‐acetate (PMA; Sigma‐Aldrich, P1585) and ionomycin (Sigma‐Aldrich, I0634) in the presence of brefeldin A (BioLegend, 420601) for 4 h. Then subsequently fixed and permeabilized BY BD fixation/permeabilization solution kit (BD Biosciences, 554714). After that, samples were cover with antibodies for 15 minutes. The details of antibodies are listed in Table S2. Flow cytometric analyses were performed using a DxFLEX flow cytometer (Beckman), and the data were analyzed using FlowJo 10 software.

### 4.10 Transmission Electron Microscopy

Cultured cells were digested, harvested and fixed by 2.5% glutaraldehyde at RT for 30 min in the dark, followed by storage at 4°C until further processing. A transmission electron microscopy (TEM) assay was performed using Servicebio (Wuhan, China).

### 4.11 Alpha Fold 3 Prediction of Structural Analysis

The architecture of protein‐promoter assemblies was computationally predicted through AlphaFold3, followed by an in‐depth characterization and visual mapping of the binding surfaces via PyMOL software

### 4.12 Cell Proliferation Assay

To evaluate T cell proliferation, CD8^+^ T cells (1×10^5^) were stained with 0.4 µm CFSE (MCE, HY‐D0938Y) and activated by anti‐CD3/CD28 beads (BioLegend) for 96 h. The result can be captured by flow cytometry.

### 4.13 Mouse Tumor Models

For tumor induction, 1 × 10^6^ LLC or LLC‐OVA tumor cells were challenged into age‐ and sex‐matched C57BL/6 hosts. One‐week post‐inoculation, mouse received an adoptive transfer with 1 × 10^6^ *Hs1bp3^−/−^ or Hs1bp3^+/+^* CD8^+^ OT‐1 T cells. To examine the effect of Sirt1 or MNAM on CD8^+^ T cells, *Hs1bp3^−/−^* OT‐1 CD45.1^+^ CD8^+^ T cells (1 × 10^5^) were treated with 0.2 µm SIRT1‐IN‐1 (MedChemExpress, HY‐136199) or 10 mm MNAM (MedChemExpress, HY‐124124) *ex vivo* for 48 h prior to adoptive transfer. Tumor size was quantified following formula: *V* = length × width^2^ × 0.5 mm^3^ at 2‐day intervals. To characterize the tumor‐infiltrating immune cell phenotype, tumor tissues were harvested 14 days post‐engraftment.

For tumor rechallenge experiments, two weeks after the mice were inoculated with tumors, the tumors were surgically removed. The mice were then challenged with 10^6^ LLC or LLC‐OVA tumor cells in the left flank.

To deplete CD8^+^ T cells in vivo, CD8‐depleting antibodies (150 µg/per; BioXcell, BE00613) were intraperitoneally injected one day before tumor challenge, followed by the consecutive injections every 2 days.

H322‐luciferase tumor cells (1 × 10^6^) were engrafted onto the flanks of NOD‐SCID recipients. 5 × 10^6^ CAR‐T cells were delivered intravenously to evaluate anti‐tumor efficacy.

### 4.14 Imaging Flow Cytometry

Intracellular cytokines, Sirt1 were staining using the BD fixation/permeabilization solution kit (BD Biosciences, 554714). Following a 25 min incubation at 4°C. Hoechst (Solarbio, IH0070) was used for nuclear staining. It was performed through ImageStream system (Amnis), and the outcomes was analyzed by IDEAS 3.0 Software.

### 4.15 Measurement of Metabolites and SIRT1 Activity

Intracellular levels of 1‐methylnicotinamide (MNAM) were quantified via a commercially available ELISA kit (CAMILO Biological). The level of NAD^+^ was measured with NAD^+^ /NADH assay Kit (Beyotime, S0175). SIRT1 enzymatic activity was assessed through a fluorometric SIRT1 activity assay kit (Abcam, ab156065) followed by manufacturer's instructions.

### 4.16 Luciferase Reporter Assay

Luciferase reporters and the pRL‐TK Renilla luciferase control plasmid, which was designed and constructed by GenePharma, were co‐transfected into Jurkat cells using different combinations of Lipofectamine 3000 (Invitrogen) in different combinations. Luciferase was quantified using a Dual‐Luciferase Reporter Assay Kit (MedChemExpress, HY‐K1013).

### 4.17 Immunofluorescence

Cells were subjected to fixation in 4% paraformaldehyde, then mixed with 0.3% Triton X‐100 (Sangon Biotech, A600198). After washing twice, cells were with the primary antibodies overnight at 4°C and then with the secondary antibodies at room temperature. Nuclei were stained with DAPI (Solarbio, C0060). Fluorescence images were acquired using a Nikon AX/AX R confocal microscope. Fluorescent signals were captured using a Nikon AX/AX R confocal system, and quantified the results using the Coloc2 plugin within the ImageJ software environment.

### 4.18 Co‐Immunoprecipitation

The 500 µL cell lysates (200‐1000 µg) were supplemented with 1–5 µg Monoclonal/polyclonal antibodies FOXO3, SIRT1 (Abclonal). And it was mixed gently for overnight at 4°C, followed by incubation with 5 µL Protein A and Protein G (absin, abs955) for 1 h. The beads were subsequently washed four times with 0.5 mL wash buffer (absin, abs955). The results were analyzed by western blot.

### 4.19 Chromatin Immunoprecipitation (ChIP) Assay

ChIP assays were performed using the SimpleChIP Enzymatic Chromatin Immunoprecipitation Kit (Cell Signaling Technology). *Hs1bp3^−/−^ or Hs1bp3^+/+^* CD8^+^ T cells were cross‐linked with 1% formaldehyde at room temperature for 10 min. Subsequently, cross‐linked chromatin DNA was disrupted by sonication. Chromatin immunoprecipitation was executed using 4 µg of Foxo3 antibody (Proteintech, 10849‐1‐AP) and protein G magnetic beads, and the samples were incubated overnight at 4°C with rotation. IgG was used as a negative control. The protein G magnetic beads were washed, chromatin was eluted from the antibody/protein G magnetic bead complex, and the cross‐links were reversed. The precipitated DNA was quantified by using qPCR. The Bcl6 promoter primer: F: 5’‐ CCTCCGTAGCTAGCATTGTG‐3’; R: 5’‐GGCAGGAGTGTTCTGTGAAA‐3’.

### 4.20 Multi‐Round Co‐Culture Killing Model

H322 cells were plated at a density of 1 × 10^5^ cells per well 24 h prior to CAR‐T cells. CAR‐T cells were co‐cultured with tumor cells at (E:T) ratio of 5:1. On day 4, cells were harvested, and residual T cells and tumor cells were tested by flow cytometry. T cells in other replicate well were collected, and added to the wells with tumor cells 1 day in advance. The above steps were repeated 4 times.

### 4.21 RNA Interference Using shRNA

To downregulate Sirt1, the following shRNA was: shSirt1: 5′‐GCCATGTTTGATATTGAGTAT‐3′, the scrambled control sequence (shNC): 5′‐CCTAAGGTTAAGTCGCCCTCG‐3′, as previous report [50]. HEK293T cells were transfected with jetPRIME reagent (Polyplus, 101000046) and 5 µg shRNAs plasmids along with 3 µg of psPAX2 and 2 µg of pMD2.G packaging mix. Lentiviral supernatants were harvested 48 h post‐transfection and used for T cell transduction.

### 4.22 Statistical Analyses

Statistical results are valued as the mean ± SEM. Significant differences between groups by paired Student's t‐test, unpaired Student's t‐test, one‐way ANOVA, log‐rank Mantel‐Cox test or two‐way ANOVA. Statistical significance was set at *p < 0.05, **p < 0.01, and ***p < 0.001, ****p < 0.0001, ns no significance present by statistical analysis conducted in GraphPad Prism.

## Author Contributions

**Jixing Fan**: conceptualization, validation, methodology, software, data curation. **Yu Ping**: conceptualization. **Siyang Wang**: conceptualization, methodology, data curation, software, formal analysis, validation, writing – original draft, writing – review and editing, visualization. **Xiaoqian Fan**: methodology, data curation, writing – original draft, validation. **Chang Yao**: conceptualization, methodology, software, data curation. **Xuan Zhao**: conceptualization. **Congcong Li**: conceptualization, methodology. **Chunyi Shen**: conceptualization, methodology. **Yifan Huang**: visualization, validation, software. **Jiqi Shan**: conceptualization, methodology. **Jinyan Liu**: conceptualization, methodology. **Zhen Zhang**: conceptualization, writing – original draft, writing – review and editing, project administration, resources, supervision. **Yi Zhang**: conceptualization, funding acquisition, writing – original draft, writing – review and editing, project administration, resources, supervision. **Shengdian Wang**: conceptualization, methodology, writing – review and editing, resources, project administration, supervision.

## Funding

This work was supported by the National Key R&D Program: Noncommunicable Chronic Diseases‐National Science and Technology Major Project (Grant no. 2025ZD0552500), Intergovernmental International Science and Technology Innovation Cooperation Project (Grant no. 2022YFE0141000), National Natural Science Foundation of China (82403844), Medical Science and Technology Project of Henan Province (262102311020), Health science and Technology Innovation Outstanding Young Talents Training Project (YQRC2023015), and the Henan Province Special Program for Clinical Research‐Oriented Physicians (HNCRD202432). We would like to thank biorender.com for providing the illustrations.

## Conflicts of Interest

The authors declare no conflicts of interest.

## Data and Code Availability

The raw data for RNA‐seq and CUT&Tag were deposited in the Genome Sequence Archive at the National Genomics Data Center, China National Center for Bioinformation (PRJCA056289). The raw data for metabolomics analysis were deposited in the OMIX, China National Center for Bioinformation/Beijing Institute of Genomics, Chinese Academy of Sciences (OMIX015525). All other data are available in the article and its Supplementary files or from the corresponding author upon request.

## Supporting information




**Supporting File**: advs77284‐sup‐0001‐SuppMat.docx.

## Data Availability

The data that support the findings of this study are openly available in BIG Sub at https://ngdc.cncb.ac.cn, reference number PRJCA056289.
