# Supplementary material for: Nicotinamide Metabolism Constrains Memory CD8+ T Cell Formation Through a Putative HS1BP3‐SIRT1‐FOXO3‐BCL6 Axis
Source: Adv Sci (Weinh). 2026 Aug 21;13(57):e77284. doi: 10.1002/advs.77284 (PMC13495949; doi:10.1002/advs.77284)
Supplement: Supplementary file 1 [file ADVS-13-e77284-s001.docx]

**Supplementary Table and Figures**

**Supplementary Table 1. Clinical characteristics of patients with lung cancer.**

| Patient. No | Sex | Age | Cancer Type | TNM |
| --- | --- | --- | --- | --- |
| 1 | Male | 55 | LUAD | T3N2M0 |
| 2 | Male | 76 | LUAD | T4N3M1 |
| 3 | Female | 74 | LUAD | pT2aN0M0 |
| 4 | Male | 67 | SCLC | T3N2M1 |
| 5 | Male | 73 | SCLC | cT1bN2bM1 |
| 6 | Female | 65 | LUAD | T1N0M0 |
| 7 | Female | 77 | LUAD | cT4N2M1c |
| 8 | Female | 70 | LUL-MIA | T1miN0M0 |

**Supplementary Table 2. Antibodies used for flow cytometry.**

| **Marker** | **Fluorochrome** | **Clone** | **Company** | **Cat#** |
| --- | --- | --- | --- | --- |
| anti-mouse CD45.1 | PE/Cyanine7 | A20 | Biolegend | 110729 |
| anti-mouse CD8 | APC/Cyanine7 | 53-6.7 | Biolegend | 100714 |
| CD69 | FITC anti-mouse | H1.2F3 | Biolegend | 104506 |
| CD28 | APC anti-mouse | 37.51 | Biolegend | 102110 |
| CD279 (PD-1) | PE anti-mouse | 29F.1A12 | Biolegend | 135206 |
| CD366  (Tim-3) | Brilliant Violet 421™ anti-mouse | B8.2C12 | Biolegend | 134019 |
| CD62L | Brilliant Violet 650™ anti-mouse | MEL-14 | Biolegend | 104453 |
| CD44 | Alexa Fluor® 700 anti-mouse/human | IM7 | Biolegend | 103025 |
| IFN-γ | PE anti-mouse | W18272D | Biolegend | 163504 |
| Granzyme B Recombinant | APC anti-human/mouse | QA16A02 | Biolegend | 372204 |
| IL-2 | Brilliant Violet 421™ anti-mouse | JES6-5H4 | Biolegend | 503825 |
| CD4 | PE/Cyanine7 anti-mouse | GK1.5 | Biolegend | 100421 |
| CD25 | FITC anti-mouse | 3C7 | Biolegend | 101907 |
| CD44 | APC anti-mouse/human | IM7 | Biolegend | 103012 |
| Annexin V | APC | — | Biolegend | 640919 |
| CD3 | PE/Cyanine7 anti-human | HIT3a | Biolegend | 300316 |
| Recombinant Protein L | FITC | — | Biolegend | 303653 |
| CD279 (PD-1) | APC anti-human | A17188A | Biolegend | 379207 |
| CD69 | Brilliant Violet 421™ anti-human | FN50 | Biolegend | 310929 |
| CD28 | Brilliant Violet 605™ anti-human | CD28.2 | Biolegend | 302967 |
| CD366  (Tim-3) | Brilliant Violet 785™ anti-human | F38-2E2 | Biolegend | 345031 |
| CD45RA | APC anti-human | HI100 | Biolegend | 304112 |
| CD62L | PE anti-human | DREG-56 | Biolegend | 304806 |
| IFN-γ | PE anti-human | W19227C | Biolegend | 383404 |
| Perforin | Percp/Cyanine5.5 anti-human | B-D48 | Biolegend | 353313 |
| Granzyme B Recombinant | APC anti-human/mouse | QA16A02 | Biolegend | 372203 |
| BCL6 | Alexa Fluor® 647 Conjugate | E5I8I | Cell Signaling Technology | 62737S |
| FoxO3a | PE Conjugate | D19A7 | Cell Signaling Technology | 14592 |
| TCF7/TCF1 | Alexa Fluor® 488 anti-Mouse | — | R&D Systems | IC8224G |
| LY108 | APC anti-mouse | 330-AJ | Biolegend | 134609 |
| Tox | PE anti-mouse/human | W17072A | Biolegend | 377122 |
| CD127 | Brilliant Violet 421™ anti-mouse | A7R34 | Biolegend | 135023 |
| Klrg1 | FITC anti-mouse/human | 2F1/KLRG1 | Biolegend | 138410 |
| Eomes | Alexa Fluor® 647 anti-mouse | W17001A | Biolegend | 157703 |
| Blimp1 | Brilliant Violet 421™ anti-mouse | 5E7 | Biolegend | 150009 |
| T-bet | APC anti-T-bet | 4B10 | Biolegend | 644813 |

**Supplementary Figure 1. HS1BP3 impairs memory CD8^+^ T cell differentiation.**

**Supplementary Figure 1. HS1BP3 impairs memory CD8^+^ T cell differentiation.**

(A) Hs1bp3 expression levels in CD8^+^ T cells derived from control and Hs1bp3 deletion OT-1 mice measured by western blotting. (B-C) The analysis of thymocytes from *Hs1bp3*^⁺/⁺^ OT-1, *Hs1bp3*^⁻/⁻^ OT-1, global *Hs1bp3*-WT *and Hs1bp3*-KO mice was performed to determine the percentage of CD4 and CD8 single-positive (SP) cells, CD4-CD8 double-positive (DP) cells, and CD4-CD8 double-negative (DN) cells within the total thymocyte population and the percentage of different stages of DN (DN1-4) cells using fluorescence-conjugated antibodies, including anti-mouse CD4, CD8, CD25 and CD44 (n = 5 biological replicates). (D) The proliferation of CD8^+^ OT-1 cells was detected using CFSE staining (n = 5 biological replicates). (E) Representative plots and bar graphs display the proportions of CD44^+^CD62L^+^ and CD44^+^CD62L^-^ subsets in the spleen of *Hs1bp3*^⁺/⁺^ and *Hs1bp3*^⁻/⁻^ OT-1 mice (n = 5 biological replicates). (F) The gating strategy of flow cytometry analysis on adoptively transferred *Hs1bp3*^⁺/⁺^ and *Hs1bp3*^⁻/⁻^ OT-1 T cells. The counting beads were used to quantify the absolute numbers of CD44^+^CD62L^+^ and CD44^+^CD62L^-^ subsets. Data are presented as mean ± SEM. Statistical significance was calculated using Student’s t test (B-D). *, *P* < 0.05; ns: no significance.

**Supplementary Figure 2. HS1BP3 deletion inhibits the expression of memory-related markers in an adoptive transfer model following *Lm*-OVA infection.**

**Supplementary Figure 2. HS1BP3 deletion inhibits the expression of memory-related markers in an adoptive transfer model following *Lm*-OVA infection.** (A) Proportions of CD127^-^KLRG1^+^ and CD127^+^ KLRG1^-^ CD8^+^ OT-1 T cells determined flow cytometry (n = 5 biological replicates). (B-C) Flow cytometry analysis of Eomes, Bcl6, Blimp-1 and T-bet expression in CD8^+^ OT-1 T cells from *Hs1bp3*^⁺/⁺^ or *Hs1bp3*^⁻/⁻^ groups (n = 5 biological replicates). Data are presented as mean ± SEM. Statistical significance was calculated using Student’s t test (A-C). *, *P* < 0.05; **, *P* < 0.01; ***, *P* < 0.001, ****, *P* < 0.0001.

**Supplementary Figure 3. HS1BP3 deletion reduces anti-tumor efficacy *in vivo*.**

**Supplementary Figure 3. HS1BP3 deletion reduces anti-tumor efficacy *in vivo*.**

(A) Experiment set up. LLC-OVA tumor cells were subcutaneously implanted into *Hs1bp3*^⁺/⁺^ and *Hs1bp3*^⁻/⁻^ OT-1 mice. CD45.1^+^CD8^+^ T cells were sorted from tumor samples on day 14. (B-C) Tumor growth of LLC-OVA lung cancer in *Hs1bp3*^⁺/⁺^ and *Hs1bp3*^⁻/⁻^ OT-1 mice (n = 5 biological replicates). (C) Survival rates of the mice (n = 5 biological replicates). (D) Frequencies and numbers of CD44^+^CD62L^+^ subset in the tumor of mice were determined using flow cytometry. Percentage of IFN-γ^+^ CD8^+^ OT-1 cells in mice. (E) Diagram of rechallenge model. (F-G) Tumor growth and survival rates of mice (n = 5 biological replicates). (H) The frequencies and numbers of CD44^+^CD62L^+^ subset in the tumor of mice flowing rechallenge (n = 5 biological replicates). IFN-γ production was determined by flow cytometry (n = 5 biological replicates). Data are presented as mean ± SEM. Statistical significance was calculated using Student’s t test (D, H). Two-way ANOVA was performed to determine the significance of tumor growth (B, F), and survival curves were analyzed using the log-rank Mantel-Cox test (C, G). *, *P* < 0.05; **, *P* < 0.01; ***, *P* < 0.001.

**Supplementary Figure 4.** **HS1BP3-mediated antitumor response is dependent on CD8⁺ T cells.**

**Supplementary Figure 4.** **HS1BP3-mediated antitumor response is dependent on CD8⁺ T cells.** (A) Schematic of the CD8⁺ T cell depletion experiment. *Hs1bp3*^⁺/⁺^ and *Hs1bp3*^⁻/⁻^ OT-1 mice were subcutaneously injected with LLC-OVA tumor cells and then treated with anti-CD8a antibody (αCD8) every 2 days starting at the indicated time points. Tumor growth was measured every 2 days and survival was recorded. (B) Flow cytometry analysis of CD8⁺ T cell depletion efficiency in blood of *Hs1bp3*^⁺/⁺^ and *Hs1bp3*^⁻/⁻^ OT-1 mice treated with or without αCD8 antibody. (C-D) Tumor growth and survival curves of LLC-OVA bearing *Hs1bp3^⁺/⁺^* and *Hs1bp3*^⁻/⁻^ OT-1 mice with or without CD8⁺ T cell depletion (n = 5 biological replicates). Data are presented as mean ± SEM. Two-way ANOVA was performed to determine the significance of tumor growth(C), and survival curves were analyzed using the log-rank Mantel-Cox test(D). **, *P* < 0.01; ****, *P* < 0.0001.

**Supplementary Figure 5. HS1BP3 deficiency promotes the expression of exhausted-associated markers.**

**Supplementary Figure 5. HS1BP3 deficiency promotes the expression of exhausted-associated markers.** (A-B) Proportions of Bcl6^+^, Ly108^+^, PD-1^+^ Tim-3^+^ and Tox^+^ tumor-infiltrating CD8^+^ OT-1 cells from LLC-OVA bearing recipients of *Hs1bp3^⁺/⁺^* and *Hs1bp3*^⁻/⁻^ OT-1 mice (n = 5 biological replicates). Data are presented as mean ± SEM. Statistical significance was calculated using Student’s t test (A-B). **, *P* < 0.01; ***, *P* < 0.001.

**Supplementary Figure 6.** **Gating strategies used for tumor CD8^+^ T cells.**

**Supplementary Figure 6.** **Gating strategies used for Tumor CD8^+^ T cells.** (A) Flowcytometry strategy for detecting CD44^+^CD62L^+^ and CD44^+^CD62L^-^cells among CD45.1^+^CD8^+^ T cells in Figure3E, 3G, 3L, 3N. (B) Representative flow cytometry staining of expression level of IFN-γ, Granzyme B, IL-2 among CD45.1^+^CD8^+^ T cells in Figure 3H, 3O.

**Supplementary Figure 7. HS1BP3 deletion contributes to the activation of SIRT1 activity.**

**Supplementary Figure 7. HS1BP3 deletion contributes to the activation of SIRT1 activity.** (A) Representative blot of CD8 and Sirt1 expression in *Hs1bp3^⁺/⁺^* and *Hs1bp3*^⁻/⁻^ OT-1 mice determined by imaging flow cytometry (Left). Quantification of Sirt1 mean fluorescence intensity (MFI) (Right) (n = 5 biological replicates). (B) Co-immunoprecipitation (Co-IP) assays were performed to detect the interaction between SIRT1 and HS1BP3. (C-D) Frequencies of CD44⁺CD62L⁺ T cells and memory-related genes expression in *Hs1bp3*^⁻/⁻^ CD8^+^ OT-1 cells treated with or without the Sirt1 inhibitor. (right, n = 5 biological replicates; left, n = 3 biological replicates). This experiment was independently performed three times. (E) Expression levels of Sirt1 in CD8^+^ OT-1 cells after Sirt1 shRNA transfection (right, n = 3 biological replicates; left, n = 5 biological replicates). This experiment was independently performed three times. (F) Frequencies of CD44⁺CD62L⁺ T cells in *Hs1bp3*^⁻/⁻^ CD8^+^ OT-1 cells treated with or without the shSirt. (n = 5 biological replicates) (G) Western blotting analysis of Sirt1 expression in *Hs1bp3*^⁻/⁻^ CD8^+^ OT-1 cells treated with or without FK866 (n = 3 biological replicates). This experiment was independently performed three times. (H) NAD⁺ levels in CD8⁺ T cells were detected (n = 5 biological replicates). (I) Frequencies of CD44^+^CD62L^+^ T cells in *Hs1bp3*^⁻/⁻^ CD8^+^ OT-1 cells treated with or without FK866 (n = 5 biological replicates). (J) Expression of memory-related genes expression in *Hs1bp3*^⁻/⁻^ CD8^+^ OT-1 cells treated with or without FK866 (n = 3 biological replicates) This experiment was independently performed three times. Data are presented as mean ± SEM. Statistical significance was calculated using Student’s t test (A, C-D, G-J) and one-way ANOVA(E-F). *, *P* < 0.05; **, *P* < 0.01; ***, *P* < 0.001; ****, *P* < 0.0001.

**Supplementary Figure 8. CD8^+^ T cell differentiation is regulated by FOXO3-BCL6 axis.**

**Supplementary Figure 8. CD8^+^ T cell differentiation is regulated by FOXO3-BCL6 axis.** (A) The expression of SIRT1, FOXO3, and HS1BP3 in Jurkat cells overexpressing HS1BP3 overexpression was analyzed by western blot (left). Immunoprecipitation against Foxo3 was performed in Jurkat cells upon treatment with HS1BP3 overexpression. The acetylated FOXO3 signal was visualized with a pan acetyl-lysine antibody (right) (n = 3 biological replicates). This experiment was independently performed three times. (B) Immunoblot and quantification of Sirt1 and Foxo3 in OVA-specific T_eff_ and T_m_ cells following Lm-OVA infection (n = 3 biological replicates). This experiment was independently performed three times. (C) Foxo3 expression in *Hs1bp3*^⁻/⁻^ CD8^+^ T cells after Foxo3 siRNA transfection (n = 3 biological replicates). This experiment was independently performed three times. (D-E) Frequencies of CD44^+^CD62L^+^ T cells and memory-related genes expression in *Hs1bp3*^⁻/⁻^ CD8^+^ OT-1 cells after Foxo3 siRNA transfection (D: n = 5 biological replicates; E: n = 3 biological replicates). This experiment was independently performed three times. (F) Expression levels of mitochondrion OXPHOS proteins in CD8^+^ OT-1 cells after Foxo3 siRNA transfection (n = 3 biological replicates). This experiment was independently performed three times. (G) Foxo3 CUT&Tag signal height, position and density heatmap within 3kb relative to the TSS for all genes in CD8⁺ T cells from *Hs1bp3*^⁺/⁺^ and *Hs1bp3*^⁻/⁻^ OT-1 mice (n = 3 biological replicates). (H) A Pie plot showing the genomic distribution of Foxo3 CUT&Tag peaks in CD8⁺ T cells from *Hs1bp3*^⁺/⁺^ and *Hs1bp3*^⁻/⁻^ OT-1 mice. (I) GO enrichment analysis of genes associated with differential Foxo3 CUT&Tag peaks. (J) Frequencies of Bcl6⁺ and Foxo3⁺ OT-1 CD8⁺ T cells from *Hs1bp3⁻/⁻* mice treated with Foxo3 siRNA alone or in combination with the Sirt1 activator (n = 5 biological replicates). (K-M) Frequencies of Bcl6⁺, Foxo3⁺ and Sirt1⁺ OT-1 CD8⁺ T cells from *Hs1bp3⁻/⁻* mice treated with Bcl6 overexpression. (n = 5 biological replicates). (N) Frequencies of CD44^+^CD62L^+^ T cells in *Hs1bp3*^⁻/⁻^ CD8^+^ OT-1 cells after Bcl6 overexpression (n = 5 biological replicates). Data are presented as mean ± SEM. Statistical significance was calculated using Student’s t test (A-F) and one-way ANOVA(J-N). *, *P* < 0.05; **, *P* < 0.01; ***, *P* < 0.001; ****, *P* < 0.0001, ns: no significance.

**Supplementary Figure 9. HS1BP3 enhances memory CAR-T cell differentiation**

**and anti-tumor function through the SIRT1-FOXO3-BCL6 axis.**

**Supplementary Figure 9. HS1BP3 enhances memory CAR-T cell differentiation**

**and anti-tumor function through the SIRT1-FOXO3-BCL6 axis.** (A) Cell numbers of CAR-T cells upon multiple rounds tumor challenge (n = 5 healthy donors). (B) Specific lysis of H322 cells after co-culture with CAR-T cells at a 5:1 (E: T) ratio and multiple rounds of tumor challenge (n = 5 healthy donors). (C) Flow cytometry analysis of CD45RA^-^CD62L^+^ subset in meso-CAR-T and HS1BP3-meso-CAR-T cells after multiple rounds of tumor challenge (n = 5 healthy donors). (D-F) Expression of SIRT1, FOXO3 and BCL6 in two CAR-T cell group (n = 5 healthy donors). Data are presented as mean ± SEM. Statistical significance was calculated using paired two-tailed Student’s t-test (A-F). *, *P* < 0.05; **, *P* < 0.01, ***, *P* < 0.001****, *P* < 0.0001.

**Supplementary Methods**

**IL-15-derived CD8^+^ T_m_ cell culture**

OT-1 CD8⁺ T cells were stimulated for 72 h using 200 ng/mL OVA257–264 peptide (SIINFEKL, MedChemExpress, 138831-86-4) and 50 IU/mL IL-2 (Gibco, 212-12-5UG). To induce T_eff_ and T_m_ cells, activated cells were then cultured in RPMI 1640 medium with either IL-2 or IL-15 (10 ng/mL; Gibco) for an additional 96 h.

**RNA-Seq**

RNA of T_eff_ and T_mem_ was obtained using TRIzol reagent (Invitrogen, 15596018), and sequenced by Jingjie PTM Biolab Co, Ltd. (China). The RNA Nano 6000 Assay Kit from a Bioanalyzer 2100 system (Agilent Technologies, Santa Clara, CA, USA) was used to evaluate the RNA integrity. The library was constructed according to the instructions of Illumina’s NEBNext® UltraTM RNA Library Prep Kit, and then the library construction and quality control of the PC12 cell samples were carried out using ordinary NEB library construction. The TruSeq PE Cluster Kit v3-cBot-HS was applied to a cBot Cluster Generation System (Illumia, San Diego, CA, USA) to cluster the index-coded samples. After clustering, 150 bp paired-end reads were generated by sequencing the library preparations on an Illumina NovaSeq platform. DEGs were p < 0.05 and the fold difference was 1.5-fold. Based on the screened DEGs, the differential genes were analyzed for GO and KEGG enrichment using ClusterProfiler.

**Liquid chromatography/mass spectrometry non-targeted metabolomics analysis**

*Hs1bp3^-/-^* and *Hs1bp3^+/+^* CD8^+^ T cells were purified by cell sorting and underwent non-targeted liquid chromatography/mass spectrometry (MS)metabolomic profiling at BIOTREE TECH (Shanghai, China). Following the completion of sequencing, the raw data were imported into the metabolomics processing software Progenesis QI (Waters Corporation, Milford, USA) to obtain a data matrix comprising retention time, mass-to-charge ratio and peak intensity. In order to reduce the errors caused by sample preparation and instrument instability, the response intensities of the sample mass spectral peaks were normalised to form a normalised data matrix. Concurrently, variables exhibiting a relative standard deviation (RSD) exceeding 30% for the quality control samples were excluded, and a log10 transformation was applied to generate the final data matrix for subsequent analysis. Furthermore, metabolite information was obtained by matching the MS and MSMS mass spectral data with the metabolic public database HMDB. The differential metabolites were subjected to principal component analysis (PCA) and orthogonal partial least squares discriminant analysis (OPLS-DA) using the R package ropls (Version 1.6.2), and the stability of the model was evaluated through seven cycles of cross-validation. The selection of significantly different metabolites was based on two criteria: the variable weight values (VIP) obtained from the OPLS-DA model and the Student’s t-test P-value. Metabolites with VIP > 1 and P < 0.05 were considered significantly different. Pathway enrichment and KEGG analyses were performed on the principal metabolites derived from disparate tissue

**Mitochondrial Respiration Assay**

Mitochondrial respiration was assessed by measuring the oxygen consumption rate (OCR) was measured using the Seahorse XF Cell Mito Stress Test Kit (Agilent Technologies, 103016-100), using an Agilent Seahorse XFe96 Analyzer (Agilent Technologies) per the manufacturer’s instructions. CD8^+^ T cells were washed in XF Base media (Seahorse XF RPMI medium with 2 mM glutamine, 10 mM glucose, 1 mM sodium pyruvate, and 5mM HEPES) before being plated onto Seahorse cell culture plates. The cells were allowed to adhere to culture plate for 1 hour and the assay media was topped to 180µL. The OCR was measured via Seahorse Mito Stress assay, with addition of oligomycin (2.0 µM), carbonyl cyanide 4 - (trifluoromethoxy) phenylhydrazone (FCCP; 1.5 µM) and Rotenone and Antimycin (1.0 μM) as the final concentration per well. The OCR Data were analyzed using Wave Software (Agilent Technologies).

**CUT&Tag and data analysis**

*Hs1bp3^-/-^ or Hs1bp3^+/+^* CD8^+^ T cells were sorted in 5 biological replicates, and sequenced by CLOUD SEQ (Shanghai, China). The CUT&Tag assay was performed as described previously with modifications. In brief, 1 ×10^5^ cells were washed gently with wash buffer (20 mM HEPES pH 7.5; 150 mM NaCl; 0.5 mM spermidine and 1× protease inhibitor cocktail) two times. Next, 10 μL concanavalin A-coated magnetic beads (Bangs Laboratories) was added to each sample and incubated at RT for 10 min. The unbound supernatant and resuspended cells were removed with Dig Wash Buffer (20 mM HEPES, pH 7.5; 150 mM NaCl; 0.5 mM spermidine; 1× protease inhibitor cocktail; 0.05% digitonin, and 2 mM EDTA), and the cells were incubated with a 1:50 dilution of an anti- Foxo3a (Cell Signaling Technology, USA) primary antibody or IgG control antibody (12–370, Millipore, USA) overnight. The cells were then incubated first with a 1:100 dilution of a secondary antibody (AP132, Millipore, USA) at RT for 60 min, subsequently with a 1:100 dilution of pA-Tn5 adapter complex at RT for 60 min and with tagmentation buffer (10 mM MgCl 2 in Dig-med Buffer) at 37 ℃ for 60 min. DNA was purified using phenolchloroformisoamyl alcohol extraction and ethanol precipitation. For library amplification, 21 μL of DNA was mixed with 2 μL of a universal i5 primer and a uniquely barcoded i7 primer. A 25 μL volume of NEBNext HiFi 2 × PCR Master Mix was added, and the sample was placed in a thermocycler with a heated lid and processed with the following thermal cycling conditions: 72 ℃ for 5 min (gap filling); 98 ℃ for 30 s; 14 cycles of 98 ℃ for 10 s and 63 ℃ for 30 s; 72 ℃ for 1 min (final extension); and holding at 8 ℃. Sequencing was performed on an Illumina NovaSeq 6000 system using 150-bp paired-end sequencing following the manufacturer’s instructions. The criteria of fold change > 2.0 (> 1 in the log_2_ ratio value) and P value < 0.05 were employed to identify differentially accessible peaks.

**Preparation of Human MESO-CAR-T cells**

The construction for the anti-mesothelin and HS1BP3 coding sequence was obtained from GeneChem (Shanghai, China). The MESO-scFv was linked to the CD28 transmembrane domain and the CD28 plus CD3zeta intracellular domains to construct a second-generation CAR. To enable co-expression of HS1BP3 alongside the CAR, the two coding sequences were separated by a P2A peptide, forming the Meso-HS1BP3 construct. HEK293T cells were plated into 10-cm cell culture dishes and cultured overnight. 1.5 mL centrifuge tubes were prepared: tube 1 was added 250 μL Opti-MEM medium (Gibco, 31985070),5 μg of main plasmid,3 μg of packing plasmid PsPAX2, and 1.5 μg of packing plasmid pMD2.G; tube 2 was added 250 μL Opti-MEM (Gibco, 31985070), and 20 μL transfection reagent jetPRIME (Polyplus, 101000046). Then, the reagent in tube 2 was added into tube 1. After gently mixing, the mixture was incubated at room temperature for 20 min and dropped into HEK293T cells. After 6 h, culture medium of HEK293T cells was replaced with fresh DMEM medium (Gibco, C11995500BT) containing 10% FBS (Biological Industries, 04-001-1ACS). Lentiviral supernatants were collected 48 h after transfection and used for T cell transduction. Lentiviral supernatant and polybrene (Sigma-Aldrich, TR-1003) were added into activated Human CD3^+^ T c-ells T cells, then T cells were centrifuged for 2 h. CAR expression and transduction efficiency were assessed by flow cytometry or western blotting.
